# Identification of hub genes and potential molecular mechanisms in gastric cancer by integrated bioinformatics analysis

**DOI:** 10.7717/peerj.5180

**Published:** 2018-07-02

**Authors:** Ling Cao, Yan Chen, Miao Zhang, De-quan Xu, Yan Liu, Tonglin Liu, Shi-xin Liu, Ping Wang

**Affiliations:** 1 Department of Radiation Oncology, Tianjin Medical University Cancer Institute and Hospital, National Clinical Research Center for Cancer; Key Laboratory of Cancer Prevention and Therapy, Tianjin; Tianjin’s Clinical Research Center for Cancer, Tianjin, Tianjin, China; 2 Department of Radiation Oncology, Cancer Hospital of Jilin Province, Changchun, Jilin, China; 3 Department of Gastrointestinal Surgery, First Hospital of Jilin University, Changchun, Jilin, China; 4 Medical Oncology Translational Research Lab, Cancer Hospital of Jilin Province, Changchun, Jilin, China; 5 Information Centre, Cancer Hospital of Jilin Province, Changchun, Jilin, China

**Keywords:** Gastric cancer, Bioinformatic analysis, Differentially expressed genes, Protein–protein interaction network, Bioinformatic mining, miRNA-gene network, KEGG pathway

## Abstract

**Objective:**

Gastric cancer (GC) is the fourth most common cause of cancer-related deaths in the world. In the current study, we aim to identify the hub genes and uncover the molecular mechanisms of GC.

**Methods:**

The expression profiles of the genes and the miRNAs were extracted from the Gene Expression Omnibus database. The identification of the differentially expressed genes (DEGs), including miRNAs, was performed by the GEO2R. Database for Annotation, Visualization and Integrated Discovery was used to perform GO and KEGG pathway enrichment analysis. The protein–protein interaction (PPI) network and miRNA-gene network were constructed using Cytoscape software. The hub genes were identified by the Molecular Complex Detection (MCODE) plugin, the CytoHubba plugin and miRNA-gene network. Then, the identified genes were verified by Kaplan–Meier plotter database and quantitative real-time PCR (qRT-PCR) in GC tissue samples.

**Results:**

A total of three mRNA expression profiles (GSE13911, GSE79973 and GSE19826) were downloaded from the Gene Expression Omnibus (GEO) database, including 69, 20 and 27cases separately. A total of 120 overlapped upregulated genes and 246 downregulated genes were identified. The majority of the DEGs were enriched in extracellular matrix organization, collagen catabolic process, collagen fibril organization and cell adhesion. In addition, three KEGG pathways were significantly enriched, including ECM-receptor interaction, protein digestion and absorption, and the focal adhesion pathways. In the PPI network, five significant modules were detected, while the genes in the modules were mainly involved in the ECM-receptor interaction and focal adhesion pathways. By combining the results of MCODE, CytoHubba and miRNA-gene network, a total of six hub genes including COL1A2, COL1A1, COL4A1, COL5A2, THBS2 and ITGA5 were chosen. The Kaplan–Meier plotter database confirmed that higher expression levels of these genes were related to lower overall survival, except for COL5A2. Experimental validation showed that the rest of the five genes had the same expression trend as predicted.

**Conclusion:**

In conclusion, COL1A2, COL1A1, COL4A1, THBS2 and ITGA5 may be potential biomarkers and therapeutic targets for GC. Moreover, ECM-receptor interaction and focal adhesion pathways play significant roles in the progression of GC.

## Introduction

As the fourth most common cancer in the world, gastric cancer (GC) is a worldwide health problem. An estimated 24,590 new GC cases and 10,720 GC related deaths occurred in the United States alone in 2015 (Siegel, Miller & Jemal, 2015). The mortality and incidence rates of GC is even higher in South America and Eastern Europe (Jemal et al., 2011). A large number of biomarkers involved in transcriptional, and post-transcriptional regulation via methylation and phosphorylation, have been implicated in cancer development (Blee, Gray & Brook, 2015). However, the findings related to GC are not consistent, and specific targets for the diagnosis and treatment of GC require confirmation (Fakhri & Lim, 2017).

Gene Expression Omnibus (GEO) database provides the opportunity for the bioinformatics mining of gene expression profiles in various cancers (Jiang & Liu, 2015). After conducting an interactive analysis on the GEO database datasets, we extracted a set of differentially expressed genes (DEGs) and miRNAs (DE miRNAs), which are potentially involved in tumorigenesis and cancer progression. In addition, we also compared the gene expression profiles of GC tissues and adjacent normal tissues. By analyzing the GO (Thomas, 2017) and KEGG pathway enrichment (Xing et al., 2016), along with the construction of protein–protein interaction (PPI) network (Khunlertgit & Yoon, 2016) and the miRNA-gene network (De Cecco et al., 2017), we identified the key genes, and the underlying signaling pathways playing a significant role in GC development.

## Materials and Methods

### Gene expression profile data

A total of three mRNA expression datasets (GSE13911, GSE79973 and GSE19826) and one miRNA expression (GSE93415) dataset were downloaded from the GEO database (https://www.ncbi.nlm.nih.gov/geo/) (Clough & Barrett, 2016). All mRNA profiles were based on the GPL570 platform (Affymetrix Human Genome U113 Plus 2.0 Array) (Agilent Technologies, Santa Clara, CA, USA). The GSE13911 dataset included 69 patients with GC, 31 non-cancerous tissues and 38 tumor tissues. The datasets of GSE79973 and GSE19826 included 10 non-cancerous tissues and 10 tumor tissues and 15 non-cancerous tissues and 12 tumor tissues respectively. In addition, the GSE93415 is based on the GPL19071 platform, and included 20 tumor tissues and 20 corresponding healthy gastric mucosal tissues.

### DEG identification

GEO2R (http://www.ncbi.nlm.nih.gov/geo/geo2r/) was used to screen DEGs and DE miRNAs between GC tissues and corresponding healthy gastric tissues. As an R programming language-based dataset analysis tool, GEO2R is based on the analysis of variance or *t*-test. Therefore, over 90% GEO datasets can be accessed and analyzed with this approach. Furthermore, GEO2R consists of a large number of experimental data types and designs, and it applies an adjusted *P*-value (adj. P) to help correct false-positives. Fold-change (FC) in gene expression was calculated with a threshold criteria of |log_2_FC| ≥ 1 and the adj. *P* < 0.05 was set for DEGs and DE miRNAs selection. Funrich Software (Version 3.0, http://funrich.org/index.html) was used to analyze the overlapping DEGs in the three datasets.

### Functional network establishment of DEG candidates

To determine the functions of the overlapping DEGs, an enrichment analysis was performed on KEGG and GO pathways using the Database for Annotation, Visualization and Integrated Discovery (DAVID) (Version 6.7, https://david.ncifcrf.gov/). DAVID is a reliable program for demonstrating and integrating biological functional annotations of proteins or genes (Dennis et al., 2003). In addition, the cutoff value for pathway screening and significant functionality was set to *P* < 0.01.

### PPI network construction and app analysis

The Search Tool for the Retrieval of Interacting Genes database (Version 10.0, http://string-db.org) was used to predict potential interactions between gene candidates at the protein level. A combined score of >0.4 (medium confidence score) was considered significant. Additionally, Cytoscape software (Version 3.4.0, http://www.cytoscape.org/) was utilized for constructing the PPI network. Degree ≥20 was set as the cutoff criterion. The Molecular Complex Detection (MCODE) app was used to analyze PPI network modules (Bandettini et al., 2012), and MCODE scores >3 and the number of nodes >5 were set as cutoff criteria with the default parameters (Degree cutoff ≥2, Node score cutoff ≥2, K-core ≥2 and Max depth = 100). DAVID was utilized to perform pathway enrichment analysis of gene modules. Finally, CytoHubba, a Cytoscape plugin, was utilized to explore PPI network hub genes; it provides a user-friendly interface to explore important nodes in biological networks and computes using eleven methods, of which MCC has a better performance in the PPI network (Chin et al., 2014).

### MiRNA-gene network construction and prognosis analysis

The DE miRNAs target genes were predicted using three established programs: TargetScan (Lewis, Burge & Bartel, 2005), miRTarBase (Chou et al., 2016) and miRDB (Wong & Wang, 2015), most popular databases of experimentally validated miRNA interactions. The genes that were predicted by all three programs were chosen as the targets of DE miRNAs and the miRNA-gene network was constructed. To identify the hub genes, we combined the results of MCODE, CytoHubba and miRNA-gene networks. The prognostic significance of the identified hub genes was analyzed using Kaplan–Meier plotter (http://kmplot.com), an online database which includes both clinical and expression data (Hou et al., 2017).

### Patients and tissue specimens

We analyzed samples from 20 GC patients who underwent tumor resection at the Department of Abdominal Surgery. Our study was approved by the Ethical Committee and Institutional Review Board of Jilin Cancer Hospital, Jilin, China (Approval number: 201711-045-01). The patients had not received any preoperative radiation or chemotherapy. GC and corresponding normal mucosa (at least 5 cm distant from the tumor edge) were immediately frozen in liquid nitrogen and stored at −80 °C until further analysis. Every specimen was anonymously handled based on ethical standards. All participants provided written informed consent and our study had full approval from the hospital’s Ethical Review Committee.

### RNA extraction and quantitative real-time PCR

Trizol reagent (Invitrogen, Carlsbad, CA, USA) was used to extract total RNA from the tissue samples according to the manufacturer’s protocol. Superscript II reverse transcriptase and random primers were used to synthesize cDNA (Toyobo, Osaka, Japan). Quantitative real-time PCR (qRT-PCR) was conducted on the ABI 7900HT Sequence Detection System with SYBR-Green dye (Applied Biosystems, Foster City, CA, USA; Toyobo, Osaka, Japan). The reaction parameters included a denaturation program (5 min at 95 °C), followed by an amplification and quantification program over 40 cycles (15 s at 95°C and 45 s at 65°C). Each sample was tested in triplicates, and each sample underwent a melting curve analysis to check for the specificity of amplification. Table 1 illustrates the primer sequences of hub genes. The expression level was determined as a ratio between the hub genes and the internal control GAPDH in the same mRNA sample, and calculated by the comparative CT method. Levels of COL1A2, COL1A1, COL4A1, THBS2 and ITGA5 expression were calculated by the 2^−ΔΔCt^ method.

**Table 1:** Primer sequences of PCR.

cDNA	Forward primer (5′–3′)	Reverse primer (5′–3′)
COL1A2	GCCCTCAAGGTTTCCAAG	CCTTCAATCCATCCAGACC
COL1A1	GACGAGACCAAGAACTGCC	CACGAGGACCAGAGGGA
COL4A1	AGGATTTCCTGGTACATCTCTG	GACATTCCACAATTCCATTTG
THBS2	GCATCAAGGATAACTGCCCCCATCT	TTCATTGAAGACATCGTCCCCATCA
ITGA5	TAATACCAGCCAGCCAGGAGTG	TGTCAAATTCAATGGGGGTGC

**Note:**

Primer sequences for five hub genes.

## Results

### Identification of overlapping DEGs

**Figure 1:**
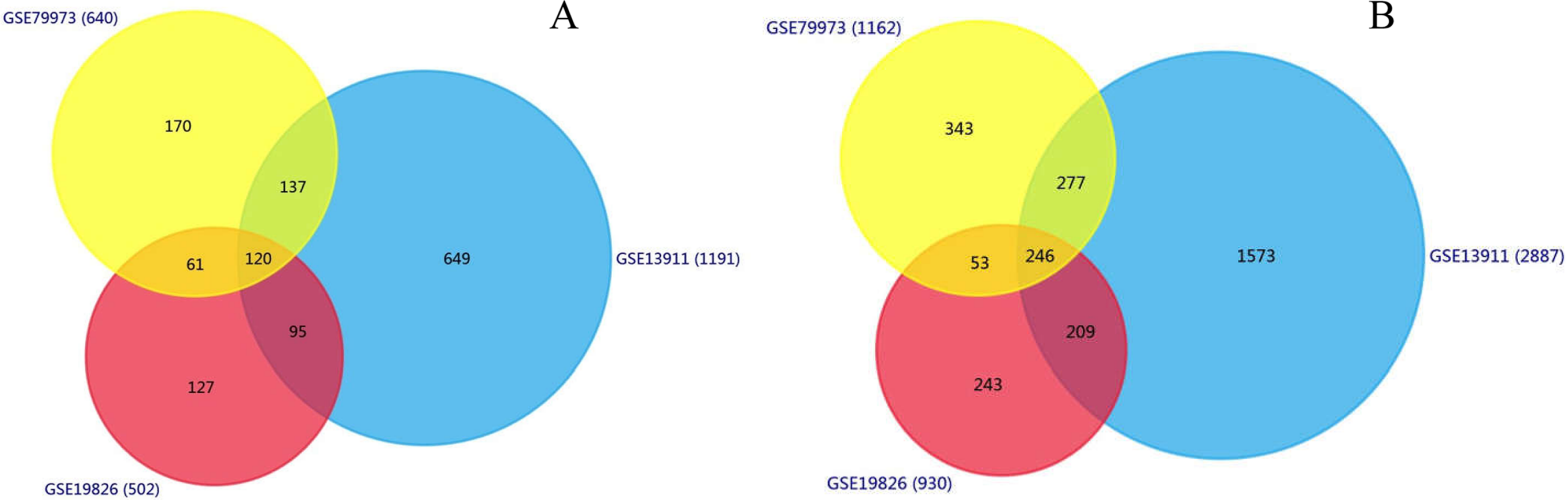
Identification of overlapping DEGs. Identification of DEGs (A) Venn diagram of 120 overlapping upregulated genes in GSE13911, GSE19826 and GSE79973; (B) Venn diagram of the 246 overlapping downregulated genes in the same datasets.

We identified 1,191, 502, 640 upregulated DEGs, and 2,887, 930, 1,162 downregulated DEGs in the GSE13911, GSE19826 and GSE79973 datasets, respectively. As shown in Fig. 1, 120 upregulated genes and 246 downregulated genes overlapped across the three datasets.

### Functional enrichment analysis of overlapped DEGs

**Figure 2:**
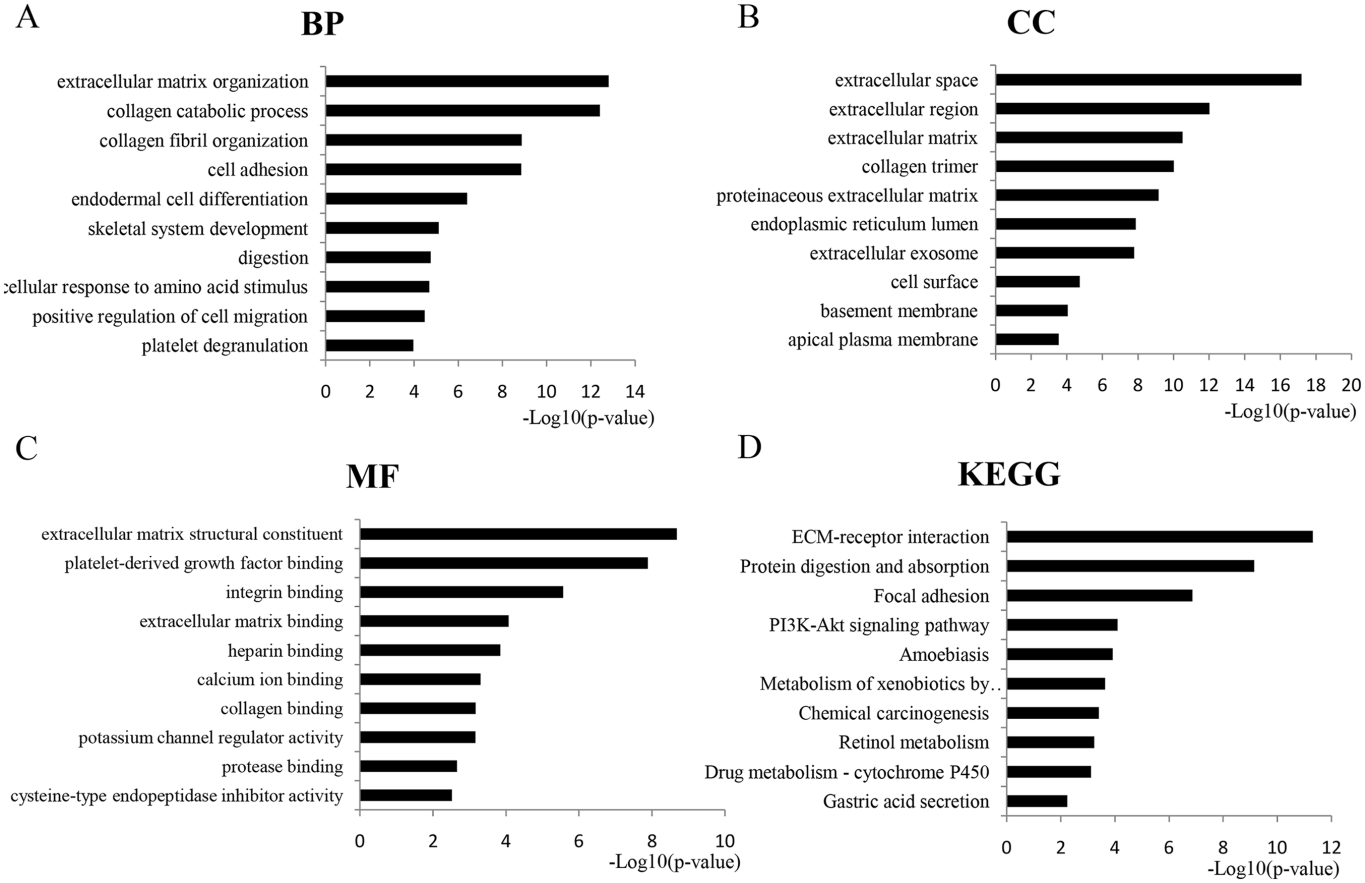
GO analysis of the overlapped DEGs. Black bars represent the number of DEGs. Here only show the top 10: (A) Biological processes (BP); (B) Cellular components (CC); (C) Molecular function (MF); (D) KEGG pathway analysis of DEGs.

GO analysis revealed 366 overlapping genes that are involved in a number of biological processes (BP), including extracellular matrix organization, collagen metabolism and fibril organization, and cell adhesion (Fig. 2A). In terms of cellular components, DEGS were mostly enriched in the extracellular space, extracellular region and the extracellular matrix (Fig. 2B). The overlapping DEGs were mainly associated with the structural organization of the extracellular matrix, platelet-derived growth factor binding, integrin binding and extracellular matrix binding in terms of molecular functions (Fig. 2C). In addition, the DEGs were enriched in three KEGG pathways, which included ECM-receptor interaction, protein digestion and absorption, focal adhesion and the PI3K-Akt signaling pathway (Fig. 2D).

### PPI network construction and analysis of modules

**Figure 3:**
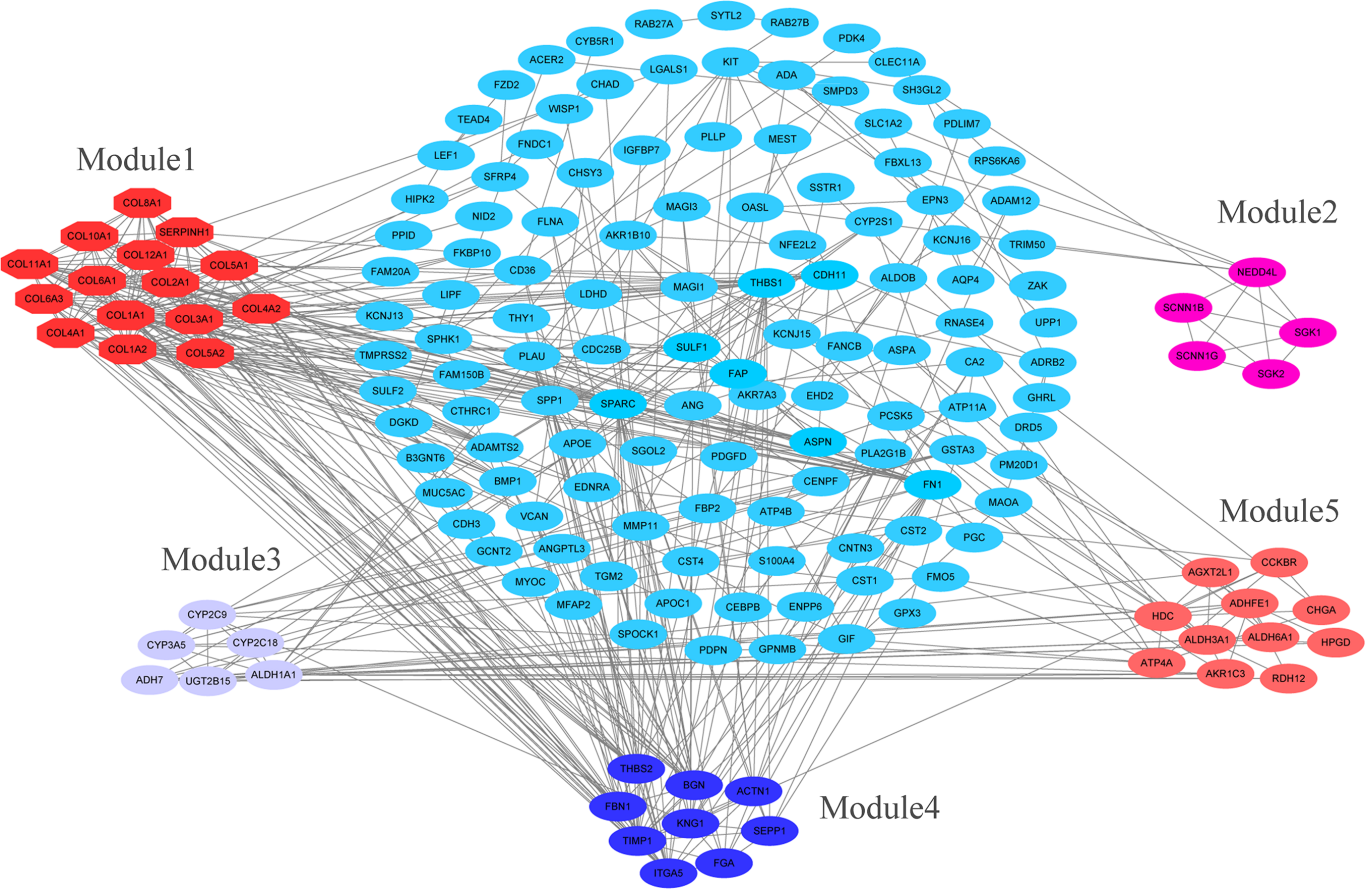
PPI network construction and module analysis. PPI network construction and module analysis. The light blue nodes in the middle represent the DEGs. The nodes of the different colors around represent the genes involved in modules and the lines represent the interaction between two nodes.

**Figure 4:**
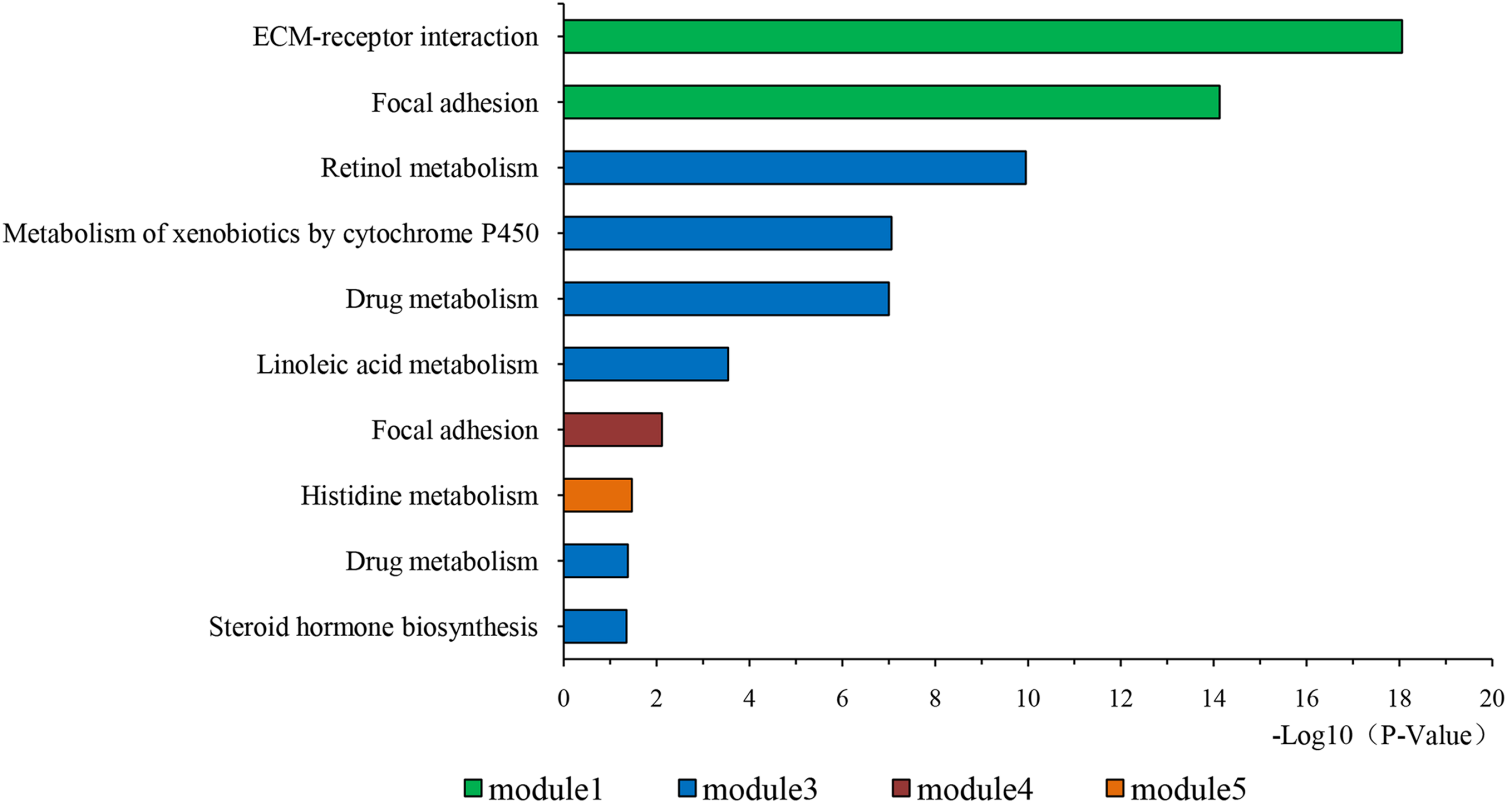
KEGG pathway of genes in five modules. KEGG pathway analysis of genes in five modules. Bars represent the number of DEGs.

**Figure 5:**
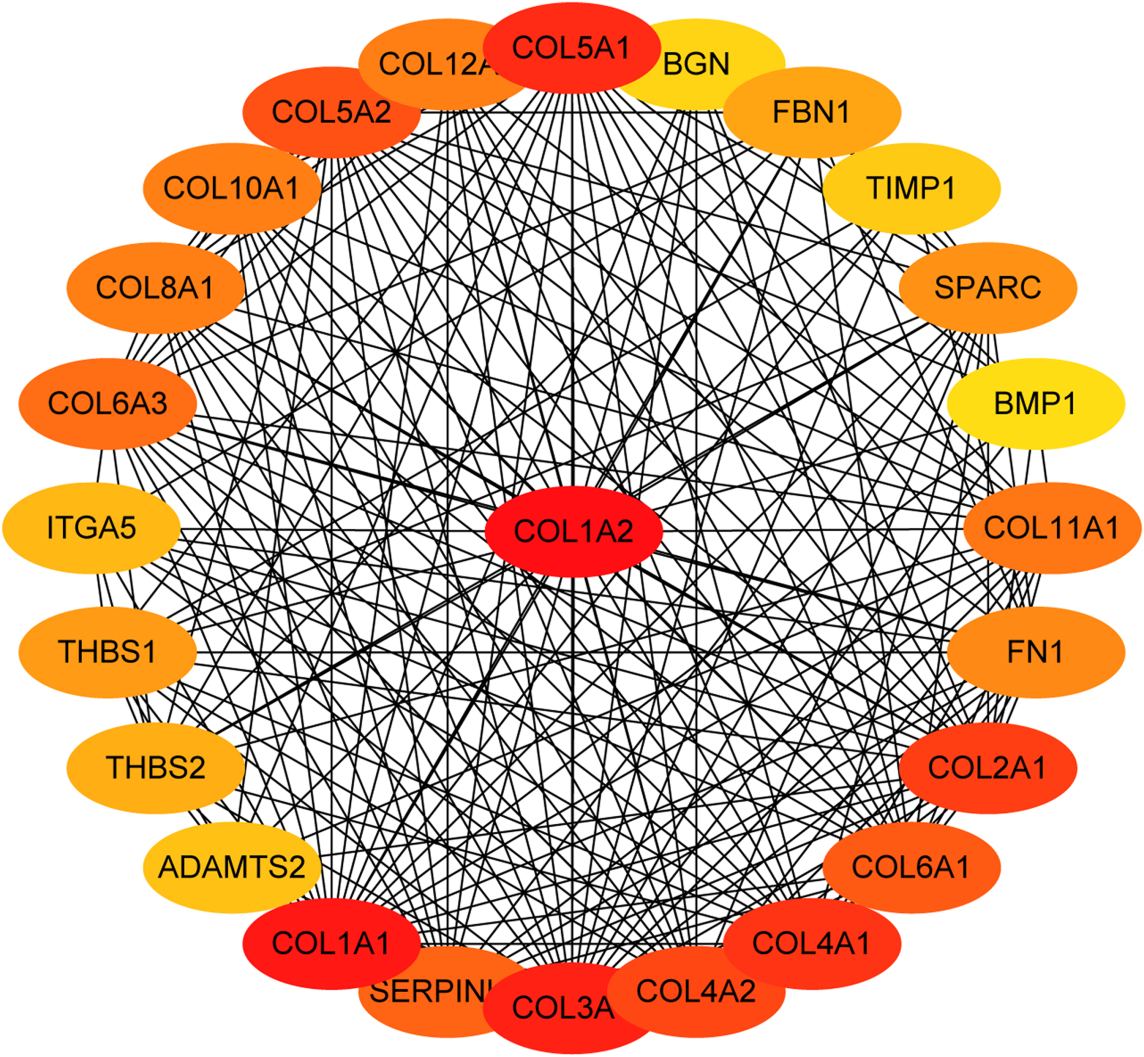
The first 25 genes network. The first 25 genes of the MMC method were chosen using CytoHubba plugin. The more forward ranking is represented by a redder color.

The overlapping DEGs indicated a distinct set of interactions and networks. The PPIs with combined scores greater than 0.4 were selected for constructing PPI networks. The entire PPI network was analyzed using MCODE, following which, five modules were chosen (Fig. 3). In addition, the KEGG pathway enrichment analysis of the module genes showed enrichment in ECM-receptor interaction and focal adhesion (Fig. 4). The first 25 genes in the MCC method were chosen by CytoHubba plugin and sequentially ordered as follows: COL1A2, COL1A1, COL3A1, COL5A1, COL4A1, COL2A1, COL4A2, COL5A2, COL6A1, SERPINH1, COL6A3, COL11A1, COL12A1, COL10A1, COL8A1, FN1, SPARC, THBS1, FBN1, THBS2, ITGA5, ADAMTS2, TIMP1, BGN, and BMP1 (Fig. 5).

### MiRNA-gene network

**Figure 6:**
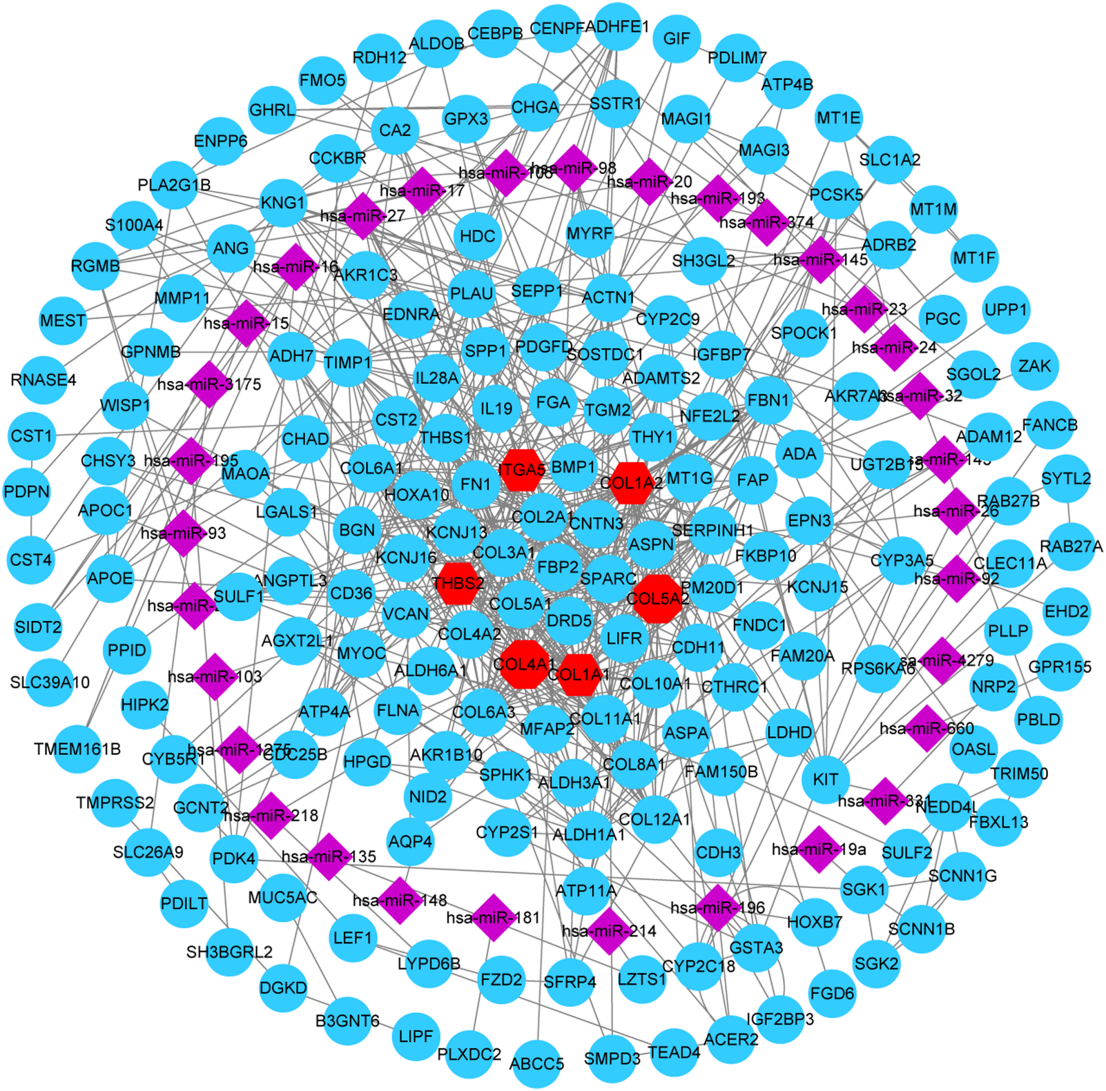
miRNA-gene network. Regulation of six hub genes in miRNA-gene network. Circular nodes stand for DEGs, red circular nodes stand for the six gene biomarkers and purple rhombus nodes represent DE miRNAs. The lines represent the regulation of relationship between two nodes.

A total of 32 DE miRNAs were identified by analyzing the miRNA expression dataset (GSE93415). Furthermore, the genes predicted by all three programs (TargetScan, miRTarBase, and miRDB) were identified as DE miRNAs target genes. In addition, to identify reliable hub genes, the target genes were compared to the DEGs and only overlapping genes were selected as the hub genes. After combining the results of MCODE, CytoHubba and miRNA-gene network, six hub genes were chosen and all of them were upregulated DEGs, including COL1A2, COL1A1, COL4A1, COL5A2, THBS2 and ITGA5 (Fig. 6).

### Kaplan–Meier survival analysis

**Figure 7:**
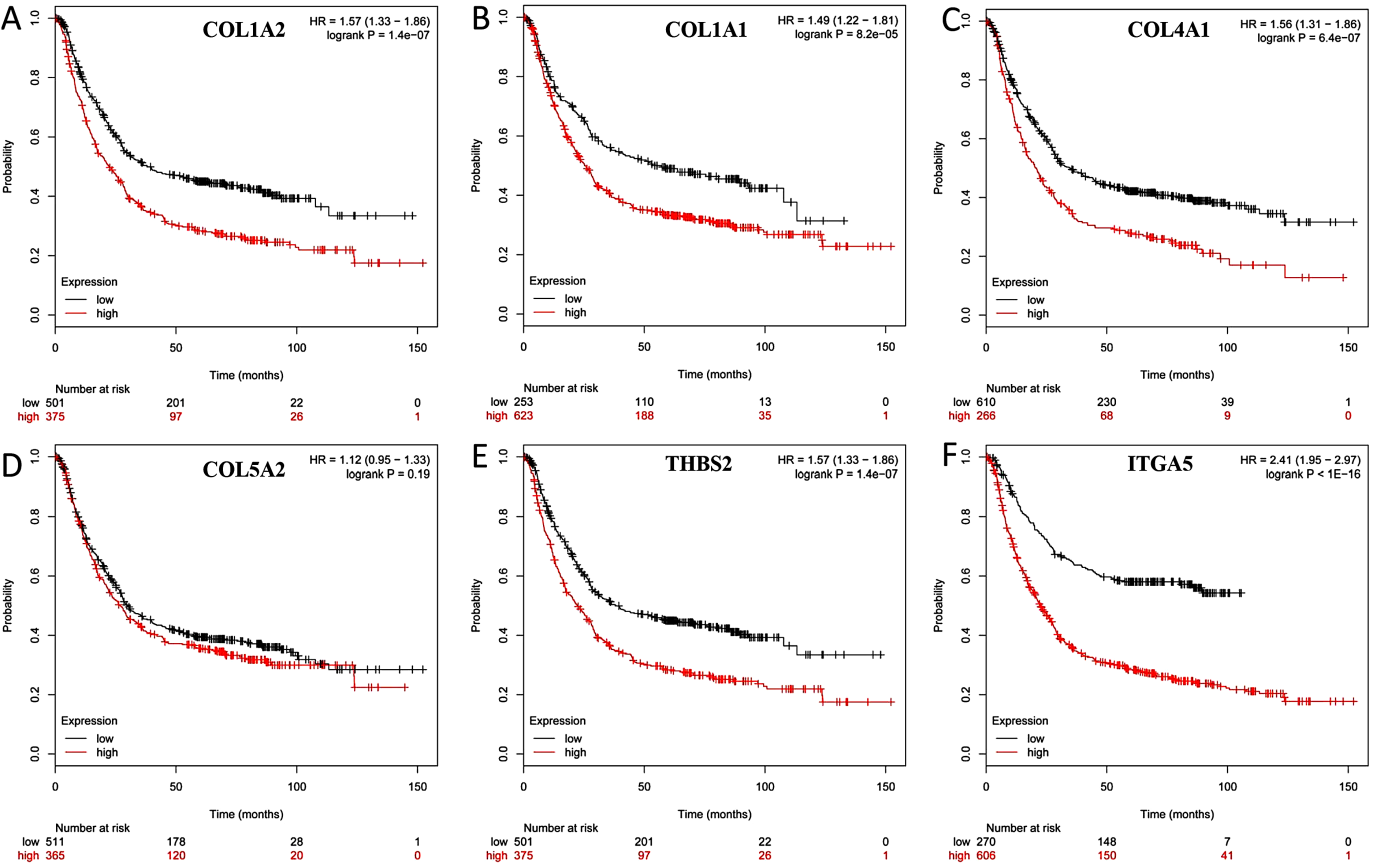
Prognostic curve of six hub genes. The prognostic significance of the hub genes in patients with GC, according to the Kaplan–Meier plotter database. (A) COL1A2; (B) COL1A1; (C) COL4A1; (D) COL5A2: (E) THBS2; (F) ITGA5. The red lines represent patients with high gene expression, and black lines represent patients with a low gene expression.

The overall survival (OS) was analyzed for 876 patients with GC using the Kaplan–Meier survival plot. Briefly, the six genes (COL1A2, COL1A1, COL4A1, COL5A2, THBS2 and ITGA5) were uploaded to the database and Kaplan–Meier curves were plotted. High expression of COL1A1 (*P* = 8.2*e*-05; adjust. *P* = 4.92*e*-04), COL1A2 (*P* = 1.4*e*-07; adjust. *P* = 8.40*e*-0.7), COL4A1 (*P* = 6.4*e*-07; adjust. *P* = 384*e*-0.6), ITGA5 (*P* < 1E-16; adjust. *P* < 9.60*e*-16), and THBS2 (*P* = 1.4*e*-07; adjust. *P* = 8.40*e*-0.7) were correlated with significantly worse OS in GC patients, while COL5A2 expression was not relevant to survival (*P* = 0.19; adjust. *P* = 1) (Fig. 7).

### The hub genes were verified within GC tissues

**Figure 8:**
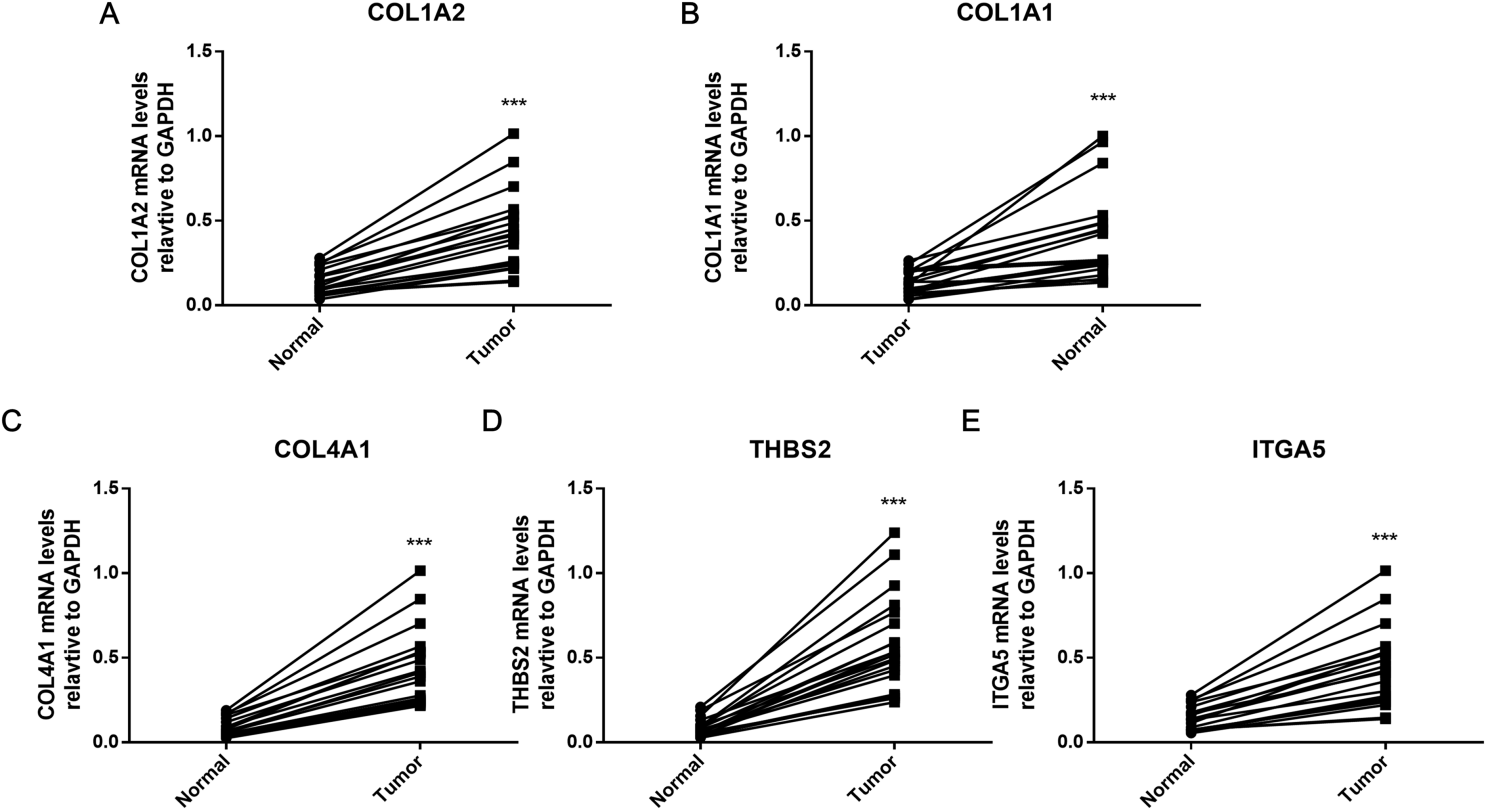
PCR results of five hub genes. Quantitative real-time PCR results for the five gene biomarkers. (A) COL1A2; (B) COL1A1; (C) COL4A1; (D) THBS2; (E) ITGA5. Expression of these DEGs was normalized against GAPDH expression. The statistical significance of differences was calculated by the Student’s *t*-test. ***P < 0.001.

To further verify the results of bioinformatics analysis, the mRNA levels of the five hub genes (COL1A2, COL1A1, COL4A1, ITGA5, and THBS2) were determined in 20 paired tumor and adjacent healthy gastric tissues with qRT-PCR. As illustrated in Fig. 8, each of the five identified genes was significantly upregulated in tumor tissue (*P* < 0.001), as predicted by the bioinformatics analysis.

## Discussion

We identified 366 overlapping DEGs—120 upregulated and 246 downregulated—in the three GC expression profile datasets. Furthermore, results from the GO analysis indicated that the overlapping genes were mostly involved in extracellular matrix organization, collagen catabolism and fibril organization, and cell adhesion at the level of BP. In addition, KEGG pathway enrichment analysis showed that the overlapping DEGs were enriched significantly within ECM-receptor interaction, protein digestion and absorption, focal adhesion pathway and PI3K-Akt signaling pathway. ECM-receptor interaction and focal adhesion were identified as the major pathways of the important modules of overlapping DEGs. These enriched pathways provide insights into the molecular mechanism of GC initiation and progression, and can therefore be useful for the development of new therapeutic strategies.

In recent years, bioinformatics analysis have been increasingly used for finding new therapeutic targets and diagnosis markers for various cancers (Jiang & Liu, 2015). Tan, Jin & Wang (2017) analyzed the potential biomarkers for prostate cancer by integrated bioinformatics analysis and identified BZRAP1-AS1 as a novel biomarker. In addition, another study (Yin, Chang & Xu, 2017) showed the vital role of the G2/M checkpoint in early hepatocellular carcinoma indicating the potential prognostic and diagnostic importance of the genes involved in the checkpoint. Similar studies have been conducted for GC as well. Sun et al. (2017) have found the core genes involved in GC by bioinformatics analysis. However, when compared to our study, their study only analyzed a profile, and only used the module method to select the gene with a high degree of connectivity. In addition, their selected genes were validated only via the Kaplan–Meier plotter database. Our study integrated three profile datasets, and then combined the results of MCODE, CytoHubba and miRNA-gene network for the identification of the hub genes. Furthermore, we verified our results by Kaplan–Meier plotter database and RT-PCR, thus increasing the reliability of our results.

We predicted five hub genes including COL1A2, COL1A1, COL4A1, THBS2 and ITGA5. Previous studies have reported some of these genes. For example, Rong et al. (2018) have reported high expression of COL1A2 in GC tissues, which was significantly correlated to the histological type and the lymph node status. Li, Ding & Li (2016) hypothesized a diagnostic use of COL1A1 to screen for early GC. They also considered COL1A1 and COL1A2 as predictors of poor clinical outcomes in GC patients. In contrast, Sun et al. (2014) showed that THBS2 expression was significantly lower in GC tissues compared to normal tissues, and that patients with higher levels of THBS2 had better prognosis. However, Zhuo et al. (2016) found higher expression of THBS2 and COL1A2 in tumor tissues and better prognosis in patients with lower THBS2 expression. Furthermore, COL4A1 and ITGA5 are not specific to GC, and studying these hub genes can therefore increase the understanding of other cancers.

To verify the results of bioinformatics analysis (Chavan, Shaughnessy & Edmondson, 2011), we used Kaplan–Meier plotter database to predict the prognostic value of these hub genes, and analyzed their expression levels in 20 pairs of GC and adjacent normal tissue samples by qRT-PCR. All hub genes, except COL5A2, were significantly correlated with worse OS for GC patients. In addition, they showed the same trend in expression as predicted by bioinformatics, thereby verifying the accuracy of our method.

## Conclusion

A total of five genes including COL1A2, COL1A1, COL4A1, THBS2 and ITGA5, were identified as GC biomarkers, and ECM-receptor interaction and focal adhesion were revealed to be important mechanisms of GC. However, our study has certain limitations such as low number of primary tissues samples, and the use of only the Kaplan–Meier plotter database to predict the prognostic value of the hub genes. The prognostic utility of these markers have to explored in GC patients and further studies need to be conducted to dissect the underlying mechanisms and related pathways of these genes.

Since cancer is a result of multiple and highly complex molecular mechanisms, a single pathway is insufficient to explain cancer pathogenesis (Zeng et al., 2013). However, our findings provide novel insights into the occurrence and progression of GC. In addition, mRNA expression profiling and the interactive network of miRNAs and mRNA are highly complex, and our bioinformatics methods are relatively new (Xu et al., 2017). Therefore, more experimental studies are necessary to confirm the present findings.

## Supplemental Information

10.7717/peerj.5180/supp-1 Supplemental Information 1 Go and kegg results.

10.7717/peerj.5180/supp-2 Supplemental Information 2 Raw data of PCR results.

10.7717/peerj.5180/supp-3 Supplemental Information 3 Raw data identification of DEGs.

10.7717/peerj.5180/supp-4 Supplemental Information 4 The miRNA results.

10.7717/peerj.5180/supp-5 Supplemental Information 5 The design of PCR primers.

## Additional Information and Declarations

### Data Availability

The following information was supplied regarding data availability: The raw data are provided in the Supplemental Files.
