# Supplementary material for: Identification of hub genes and potential molecular mechanisms in gastric cancer by integrated bioinformatics analysis
Source: PeerJ. 2018 Jul 2;6:e5180. doi: 10.7717/peerj.5180 (PMC6033081; doi:10.7717/peerj.5180)
Supplement: Supplemental Information 5 [file peerj-06-5180-s005.docx]

Design of PCR primers

COL1A1

ATGTTCAGCTTTGTGGACCTCCGGCTCCTGCTCCTCTTAGCGGCCACCGCCCTCCTGACGCACGGCCAAGAGGAAGGCCAAGTCGAGGGCCAAGACGAAGACATCCCACCAATCACCTGCGTACAGAACGGCCTCAGGTACCATGACCGAGACGTGTGGAAACCCGAGCCCTGCCGGATCTGCGTCTGCGACAACGGCAAGGTGTTGTGCGATGACGTGATCTGTGACGAGACCAAGAACTGCCCCGGCGCCGAAGTCCCCGAGGGCGAGTGCTGTCCCGTCTGCCCCGACGGCTCAGAGTCACCCACCGACCAAGAAACCACCGGCGTCGAGGGACCCAAGGGAGACACTGGCCCCCGAGGCCCAAGGGGACCCGCAGGCCCCCCTGGCCGAGATGGCATCCCTGGACAGCCTGGACTTCCCGGACCCCCCGGACCCCCCGGACCTCCCGGACCCCCTGGCCTCGGAGGAAACTTTGCTCCCCAGCTGTCTTATGGCTATGATGAGAAATCAACCGGAGGAATTTCCGTGCCTGGCCCCATGGGTCCCTCTGGTCCTCGTGGTCTCCCTGGCCCCCCTGGTGCACCTGGTCCCCAAGGCTTCCAAGGTCCCCCTGGTGAGCCTGGCGAGCCTGGAGCTTCAGGTCCCATGGGTCCCCGAGGTCCCCCAGGTCCCCCTGGAAAGAATGGAGATGATGGGGAAGCTGGAAAACCTGGTCGTCCTGGTGAGCGTGGGCCTCCTGGGCCTCAGGGTGCTCGAGGATTGCCCGGAACAGCTGGCCTCCCTGGAATGAAGGGACACAGAGGTTTCAGTGGTTTGGATGGTGCCAAGGGAGATGCTGGTCCTGCTGGTCCTAAGGGTGAGCCTGGCAGCCCTGGTGAAAATGGAGCTCCTGGTCAGATGGGCCCCCGTGGCCTGCCTGGTGAGAGAGGTCGCCCTGGAGCCCCTGGCCCTGCTGGTGCTCGTGGAAATGATGGTGCTACTGGTGCTGCCGGGCCCCCTGGTCCCACCGGCCCCGCTGGTCCTCCTGGCTTCCCTGGTGCTGTTGGTGCTAAGGGTGAAGCTGGTCCCCAAGGGCCCCGAGGCTCTGAAGGTCCCCAGGGTGTGCGTGGTGAGCCTGGCCCCCCTGGCCCTGCTGGTGCTGCTGGCCCTGCTGGAAACCCTGGTGCTGATGGACAGCCTGGTGCTAAAGGTGCCAATGGTGCTCCTGGTATTGCTGGTGCTCCTGGCTTCCCTGGTGCCCGAGGCCCCTCTGGACCCCAGGGCCCCGGCGGCCCTCCTGGTCCCAAGGGTAACAGCGGTGAACCTGGTGCTCCTGGCAGCAAAGGAGACACTGGTGCTAAGGGAGAGCCTGGCCCTGTTGGTGTTCAAGGACCCCCTGGCCCTGCTGGAGAGGAAGGAAAGCGAGGAGCTCGAGGTGAACCCGGACCCACTGGCCTGCCCGGACCCCCTGGCGAGCGTGGTGGACCTGGTAGCCGTGGTTTCCCTGGCGCAGATGGTGTTGCTGGTCCCAAGGGTCCCGCTGGTGAACGTGGTTCTCCTGGCCCTGCTGGCCCCAAAGGATCTCCTGGTGAAGCTGGTCGTCCCGGTGAAGCTGGTCTGCCTGGTGCCAAGGGTCTGACTGGAAGCCCTGGCAGCCCTGGTCCTGATGGCAAAACTGGCCCCCCTGGTCCCGCCGGTCAAGATGGTCGCCCCGGACCCCCAGGCCCACCTGGTGCCCGTGGTCAGGCTGGTGTGATGGGATTCCCTGGACCTAAAGGTGCTGCTGGAGAGCCCGGCAAGGCTGGAGAGCGAGGTGTTCCCGGACCCCCTGGCGCTGTCGGTCCTGCTGGCAAAGATGGAGAGGCTGGAGCTCAGGGACCCCCTGGCCCTGCTGGTCCCGCTGGCGAGAGAGGTGAACAAGGCCCTGCTGGCTCCCCCGGATTCCAGGGTCTCCCTGGTCCTGCTGGTCCTCCAGGTGAAGCAGGCAAACCTGGTGAACAGGGTGTTCCTGGAGACCTTGGCGCCCCTGGCCCCTCTGGAGCAAGAGGCGAGAGAGGTTTCCCTGGCGAGCGTGGTGTGCAAGGTCCCCCTGGTCCTGCTGGTCCCCGAGGGGCCAACGGTGCTCCCGGCAACGATGGTGCTAAGGGTGATGCTGGTGCCCCTGGAGCTCCCGGTAGCCAGGGCGCCCCTGGCCTTCAGGGAATGCCTGGTGAACGTGGTGCAGCTGGTCTTCCAGGGCCTAAGGGTGACAGAGGTGATGCTGGTCCCAAAGGTGCTGATGGCTCTCCTGGCAAAGATGGCGTCCGTGGTCTGACTGGCCCCATTGGTCCTCCTGGCCCTGCTGGTGCCCCTGGTGACAAGGGTGAAAGTGGTCCCAGCGGCCCTGCTGGTCCCACTGGAGCTCGTGGTGCCCCCGGAGACCGTGGTGAGCCTGGTCCCCCCGGCCCTGCTGGCTTTGCTGGCCCCCCTGGTGCTGACGGCCAACCTGGTGCTAAAGGCGAACCTGGTGATGCTGGTGCTAAAGGCGATGCTGGTCCCCCTGGCCCTGCCGGACCCGCTGGACCCCCTGGCCCCATTGGTAATGTTGGTGCTCCTGGAGCCAAAGGTGCTCGCGGCAGCGCTGGTCCCCCTGGTGCTACTGGTTTCCCTGGTGCTGCTGGCCGAGTCGGTCCTCCTGGCCCCTCTGGAAATGCTGGACCCCCTGGCCCTCCTGGTCCTGCTGGCAAAGAAGGCGGCAAAGGTCCCCGTGGTGAGACTGGCCCTGCTGGACGTCCTGGTGAAGTTGGTCCCCCTGGTCCCCCTGGCCCTGCTGGCGAGAAAGGATCCCCTGGTGCTGATGGTCCTGCTGGTGCTCCTGGTACTCCCGGGCCTCAAGGTATTGCTGGACAGCGTGGTGTGGTCGGCCTGCCTGGTCAGAGAGGAGAGAGAGGCTTCCCTGGTCTTCCTGGCCCCTCTGGTGAACCTGGCAAACAAGGTCCCTCTGGAGCAAGTGGTGAACGTGGTCCCCCTGGTCCCATGGGCCCCCCTGGATTGGCTGGACCCCCTGGTGAATCTGGACGTGAGGGGGCTCCTGGTGCCGAAGGTTCCCCTGGACGAGACGGTTCTCCTGGCGCCAAGGGTGACCGTGGTGAGACCGGCCCCGCTGGACCCCCTGGTGCTCCTGGTGCTCCTGGTGCCCCTGGCCCCGTTGGCCCTGCTGGCAAGAGTGGTGATCGTGGTGAGACTGGTCCTGCTGGTCCCGCCGGTCCTGTCGGCCCTGTTGGCGCCCGTGGCCCCGCCGGACCCCAAGGCCCCCGTGGTGACAAGGGTGAGACAGGCGAACAGGGCGACAGAGGCATAAAGGGTCACCGTGGCTTCTCTGGCCTCCAGGGTCCCCCTGGCCCTCCTGGCTCTCCTGGTGAACAAGGTCCCTCTGGAGCCTCTGGTCCTGCTGGTCCCCGAGGTCCCCCTGGCTCTGCTGGTGCTCCTGGCAAAGATGGACTCAACGGTCTCCCTGGCCCCATTGGGCCCCCTGGTCCTCGCGGTCGCACTGGTGATGCTGGTCCTGTTGGTCCCCCCGGCCCTCCTGGACCTCCTGGTCCCCCTGGTCCTCCCAGCGCTGGTTTCGACTTCAGCTTCCTGCCCCAGCCACCTCAAGAGAAGGCTCACGATGGTGGCCGCTACTACCGGGCTGATGATGCCAATGTGGTTCGTGACCGTGACCTCGAGGTGGACACCACCCTCAAGAGCCTGAGCCAGCAGATCGAGAACATCCGGAGCCCAGAGGGCAGCCGCAAGAACCCCGCCCGCACCTGCCGTGACCTCAAGATGTGCCACTCTGACTGGAAGAGTGGAGAGTACTGGATTGACCCCAACCAAGGCTGCAACCTGGATGCCATCAAAGTCTTCTGCAACATGGAGACTGGTGAGACCTGCGTGTACCCCACTCAGCCCAGTGTGGCCCAGAAGAACTGGTACATCAGCAAGAACCCCAAGGACAAGAGGCATGTCTGGTTCGGCGAGAGCATGACCGATGGATTCCAGTTCGAGTATGGCGGCCAGGGCTCCGACCCTGCCGATGTGGCCATCCAGCTGACCTTCCTGCGCCTGATGTCCACCGAGGCCTCCCAGAACATCACCTACCACTGCAAGAACAGCGTGGCCTACATGGACCAGCAGACTGGCAACCTCAAGAAGGCCCTGCTCCTCCAGGGCTCCAACGAGATCGAGATCCGCGCCGAGGGCAACAGCCGCTTCACCTACAGCGTCACTGTCGATGGCTGCACGAGTCACACCGGAGCCTGGGGCAAGACAGTGATTGAATACAAAACCACCAAGACCTCCCGCCTGCCCATCATCGATGTGGCCCCCTTGGACGTTGGTGCCCCAGACCAGGAATTCGGCTTCGACGTTGGCCCTGTCTGCTTCCTGTAA

F GACGAGACCAAGAACTGCC

R CACGAGGACCAGAGGGA

337bp 55-65℃


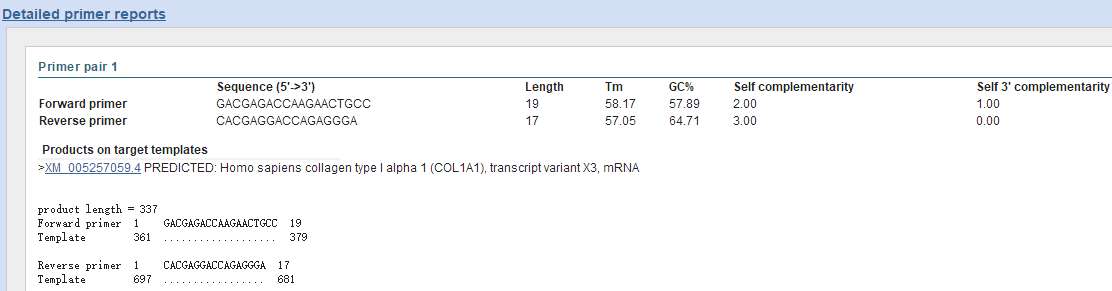


COL1A2

ATGCTCAGCTTTGTGGATACGCGGACTTTGTTGCTGCTTGCAGTAACCTTATGCCTAGCAACATGCCAATCTTTACAAGAGGAAACTGTAAGAAAGGGCCCAGCCGGAGATAGAGGACCACGTGGAGAAAGGGGTCCACCAGGCCCCCCAGGCAGAGATGGTGAAGATGGTCCCACAGGCCCTCCTGGTCCACCTGGTCCTCCTGGCCCCCCTGGTCTCGGTGGGAACTTTGCTGCTCAGTATGATGGAAAAGGAGTTGGACTTGGCCCTGGACCAATGGGCTTAATGGGACCTAGAGGCCCACCTGGTGCAGCTGGAGCCCCAGGCCCTCAAGGTTTCCAAGGACCTGCTGGTGAGCCTGGTGAACCTGGTCAAACTGGTCCTGCAGGTGCTCGTGGTCCAGCTGGCCCTCCTGGCAAGGCTGGTGAAGATGGTCACCCTGGAAAACCCGGACGACCTGGTGAGAGAGGAGTTGTTGGACCACAGGGTGCTCGTGGTTTCCCTGGAACTCCTGGACTTCCTGGCTTCAAAGGCATTAGGGGACACAATGGTCTGGATGGATTGAAGGGACAGCCCGGTGCTCCTGGTGTGAAGGGTGAACCTGGTGCCCCTGGTGAAAATGGAACTCCAGGTCAAACAGGAGCCCGTGGGCTTCCTGGTGAGAGAGGACGTGTTGGTGCCCCTGGCCCAGCTGGTGCCCGTGGCAGTGATGGAAGTGTGGGTCCCGTGGGTCCTGCTGGTCCCATTGGGTCTGCTGGCCCTCCAGGCTTCCCAGGTGCCCCTGGCCCCAAGGGTGAAATTGGAGCTGTTGGTAACGCTGGTCCTGCTGGTCCCGCCGGTCCCCGTGGTGAAGTGGGTCTTCCAGGCCTCTCCGGCCCCGTTGGACCTCCTGGTAATCCTGGAGCAAACGGCCTTACTGGTGCCAAGGGTGCTGCTGGCCTTCCCGGCGTTGCTGGGGCTCCCGGCCTCCCTGGACCCCGCGGTATTCCTGGCCCTGTTGGTGCTGCCGGTGCTACTGGTGCCAGAGGACTTGTTGGTGAGCCTGGTCCAGCTGGCTCCAAAGGAGAGAGCGGTAACAAGGGTGAGCCCGGCTCTGCTGGGCCCCAAGGTCCTCCTGGTCCCAGTGGTGAAGAAGGAAAGAGAGGCCCTAATGGGGAAGCTGGATCTGCCGGCCCTCCAGGACCTCCTGGGCTGAGAGGTAGTCCTGGTTCTCGTGGTCTTCCTGGAGCTGATGGCAGAGCTGGCGTCATGGGCCCTCCTGGTAGTCGTGGTGCAAGTGGCCCTGCTGGAGTCCGAGGACCTAATGGAGATGCTGGTCGCCCTGGGGAGCCTGGTCTCATGGGACCCAGAGGTCTTCCTGGTTCCCCTGGAAATATCGGCCCCGCTGGAAAAGAAGGTCCTGTCGGCCTCCCTGGCATCGACGGCAGGCCTGGCCCAATTGGCCCAGCTGGAGCAAGAGGAGAGCCTGGCAACATTGGATTCCCTGGACCCAAAGGCCCCACTGGTGATCCTGGCAAAAACGGTGATAAAGGTCATGCTGGTCTTGCTGGTGCTCGGGGTGCTCCAGGTCCTGATGGAAACAATGGTGCTCAGGGACCTCCTGGACCACAGGGTGTTCAAGGTGGAAAAGGTGAACAGGGTCCCCCTGGTCCTCCAGGCTTCCAGGGTCTGCCTGGCCCCTCAGGTCCCGCTGGTGAAGTTGGCAAACCAGGAGAAAGGGGTCTCCATGGTGAGTTTGGTCTCCCTGGTCCTGCTGGTCCAAGAGGGGAACGCGGTCCCCCAGGTGAGAGTGGTGCTGCCGGTCCTACTGGTCCTATTGGAAGCCGAGGTCCTTCTGGACCCCCAGGGCCTGATGGAAACAAGGGTGAACCTGGTGTGGTTGGTGCTGTGGGCACTGCTGGTCCATCTGGTCCTAGTGGACTCCCAGGAGAGAGGGGTGCTGCTGGCATACCTGGAGGCAAGGGAGAAAAGGGTGAACCTGGTCTCAGAGGTGAAATTGGTAACCCTGGCAGAGATGGTGCTCGTGGTGCTCCTGGTGCTGTAGGTGCCCCTGGTCCTGCTGGAGCCACAGGTGACCGGGGCGAAGCTGGGGCTGCTGGTCCTGCTGGTCCTGCTGGTCCTCGGGGAAGCCCTGGTGAACGTGGTGAGGTCGGTCCTGCTGGCCCCAATGGATTTGCTGGTCCTGCTGGTGCTGCTGGTCAACCTGGTGCTAAAGGAGAAAGAGGAGCCAAAGGGCCTAAGGGTGAAAACGGTGTTGTTGGTCCCACAGGCCCCGTTGGAGCTGCTGGCCCAGCTGGTCCAAATGGTCCCCCCGGTCCTGCTGGAAGTCGTGGTGATGGAGGCCCCCCTGGTATGACTGGTTTCCCTGGTGCTGCTGGACGGACTGGTCCCCCAGGACCCTCTGGTATTTCTGGCCCTCCTGGTCCCCCTGGTCCTGCTGGGAAAGAAGGGCTTCGTGGTCCTCGTGGTGACCAAGGTCCAGTTGGCCGAACTGGAGAAGTAGGTGCAGTTGGTCCCCCTGGCTTCGCTGGTGAGAAGGGTCCCTCTGGAGAGGCTGGTACTGCTGGACCTCCTGGCACTCCAGGTCCTCAGGGTCTTCTTGGTGCTCCTGGTATTCTGGGTCTCCCTGGCTCGAGAGGTGAACGTGGTCTACCAGGTGTTGCTGGTGCTGTGGGTGAACCTGGTCCTCTTGGCATTGCCGGCCCTCCTGGGGCCCGTGGTCCTCCTGGTGCTGTGGGTAGTCCTGGAGTCAACGGTGCTCCTGGTGAAGCTGGTCGTGATGGCAACCCTGGGAACGATGGTCCCCCAGGTCGCGATGGTCAACCCGGACACAAGGGAGAGCGCGGTTACCCTGGCAATATTGGTCCCGTTGGTGCTGCAGGTGCACCTGGTCCTCATGGCCCCGTGGGTCCTGCTGGCAAACATGGAAACCGTGGTGAAACTGGTCCTTCTGGTCCTGTTGGTCCTGCTGGTGCTGTTGGCCCAAGAGGTCCTAGTGGCCCACAAGGCATTCGTGGCGATAAGGGAGAGCCCGGTGAAAAGGGGCCCAGAGGTCTTCCTGGCTTAAAGGGACACAATGGATTGCAAGGTCTGCCTGGTATCGCTGGTCACCATGGTGATCAAGGTGCTCCTGGCTCCGTGGGTCCTGCTGGTCCTAGGGGCCCTGCTGGTCCTTCTGGCCCTGCTGGAAAAGATGGTCGCACTGGACATCCTGGTACAGTTGGACCTGCTGGCATTCGAGGCCCTCAGGGTCACCAAGGCCCTGCTGGCCCCCCTGGTCCCCCTGGCCCTCCTGGACCTCCAGGTGTAAGCGGTGGTGGTTATGACTTTGGTTACGATGGAGACTTCTACAGGGCTGACCAGCCTCGCTCAGCACCTTCTCTCAGACCCAAGGACTATGAAGTTGATGCTACTCTGAAGTCTCTCAACAACCAGATTGAGACCCTTCTTACTCCTGAAGGCTCTAGAAAGAACCCAGCTCGCACATGCCGTGACTTGAGACTCAGCCACCCAGAGTGGAGCAGTGGTTACTACTGGATTGACCCTAACCAAGGATGCACTATGGATGCTATCAAAGTATACTGTGATTTCTCTACTGGCGAAACCTGTATCCGGGCCCAACCTGAAAACATCCCAGCCAAGAACTGGTATAGGAGCTCCAAGGACAAGAAACACGTCTGGCTAGGAGAAACTATCAATGCTGGCAGCCAGTTTGAATATAATGTAGAAGGAGTGACTTCCAAGGAAATGGCTACCCAACTTGCCTTCATGCGCCTGCTGGCCAACTATGCCTCTCAGAACATCACCTACCACTGCAAGAACAGCATTGCATACATGGATGAGGAGACTGGCAACCTGAAAAAGGCTGTCATTCTACAGGGCTCTAATGATGTTGAACTTGTTGCTGAGGGCAACAGCAGGTTCACTTACACTGTTCTTGTAGATGGCTGCTCTAAAAAGACAAATGAATGGGGAAAGACAATCATTGAATACAAAACAAATAAGCCATCACGCCTGCCCTTCCTTGATATTGCACCTTTGGACATCGGTGGTGCTGACCAGGAATTCTTTGTGGACATTGGCCCAGTCTGTTTCAAATAA

F GCCCTCAAGGTTTCCAAG

R CCTTCAATCCATCCAGACC

243bp 50-60℃


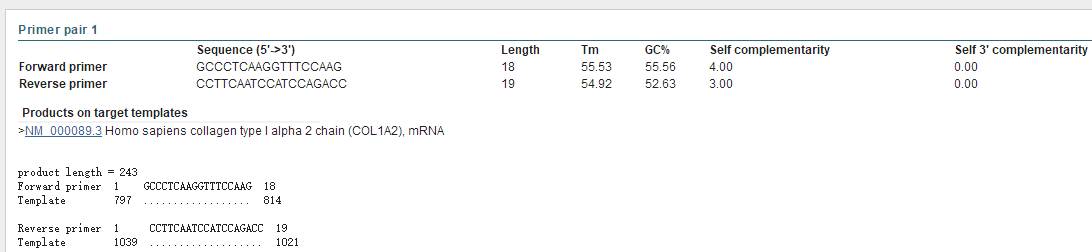


COL5A2

ATGATGGCAAACTGGGCGGAAGCAAGACCTCTCCTCATTCTTATTGTTTTATTAGGGCAATTTGTCTCAATAAAAGCCCAGGAAGAAGACGAGGATGAAGGATATGGTGAAGAAATAGCCTGCACTCAGAATGGCCAGATGTACTTAAACAGGGACATTTGGAAACCTGCCCCTTGTCAGATCTGTGTCTGTGACAATGGAGCCATTCTCTGTGACAAGATAGAATGCCAGGATGTGCTGGACTGTGCCGACCCTGTAACGCCCCCTGGGGAATGCTGTCCTGTCTGTTCACAAACACCTGGAGGTGGCAATACCAATTTTGGTAGAGGAAGAAAGGGACAAAAGGGAGAACCAGGATTAGTGCCTGTTGTAACAGGCATACGTGGTCGTCCAGGACCGGCAGGACCTCCAGGATCACAGGGACCAAGAGGAGAGCGAGGGCCAAAAGGAAGACCTGGCCCTCGTGGACCTCAGGGAATTGATGGAGAACCAGGTGTTCCTGGTCAACCTGGTGCTCCAGGACCTCCTGGACATCCGTCCCACCCAGGACCCGATGGCTTGAGCAGGCCGTTTTCAGCTCAAATGGCTGGGTTGGATGAAAAATCTGGACTTGGGAGTCAAGTAGGACTAATGCCTGGCTCTGTGGGTCCTGTTGGCCCAAGGGGACCACAGGGTTTACAAGGACAGCAAGGTGGTGCAGGACCTACAGGACCTCCTGGTGAACCTGGTGATCCTGGACCAATGGGTCCGATTGGTTCACGTGGACCAGAGGGCCCTCCTGGTAAACCTGGGGAAGATGGTGAACCTGGCAGAAATGGAAATCCTGGTGAAGTGGGATTTGCAGGATCTCCGGGAGCTCGTGGATTTCCTGGGGCTCCTGGTCTTCCAGGTCTGAAGGGTCACCGAGGACACAAAGGTCTTGAAGGCCCTAAAGGTGAAGTTGGAGCACCTGGTTCCAAGGGTGAAGCTGGCCCCACTGGTCCAATGGGTGCCATGGGTCCTCTGGGTCCGAGGGGAATGCCAGGAGAGAGAGGGAGACTTGGGCCACAGGGTGCTCCTGGACAACGAGGTGCACATGGTATGCCTGGAAAACCTGGACCAATGGGTCCTCTTGGGATACCAGGCTCTTCTGGTTTTCCAGGAAATCCTGGAATGAAGGGAGAAGCAGGTCCTACAGGGGCGCGAGGCCCTGAAGGTCCTCAGGGGCAGAGAGGTGAAACTGGGCCCCCAGGTCCAGTTGGCTCTCCAGGTCTTCCTGGTGCAATAGGAACTGATGGTACTCCTGGTGCCAAAGGCCCAACGGGCTCTCCAGGTACCTCTGGTCCTCCTGGCTCAGCAGGGCCTCCTGGATCTCCAGGACCTCAGGGTAGCACTGGTCCTCAGGGAATTCGAGGCCAACCGGGTGATCCAGGAGTTCCAGGTTTCAAAGGAGAAGCTGGCCCAAAAGGGGAACCAGGGCCACATGGTATTCAGGGTCCGATAGGCCCACCCGGTGAAGAAGGCAAAAGAGGTCCCAGAGGTGACCCAGGAACAGTTGGTCCTCCAGGGCCAGTGGGAGAAAGGGGTGCTCCTGGCAATCGTGGTTTTCCAGGCTCTGATGGTTTACCTGGGCCAAAGGGTGCTCAAGGAGAACGGGGTCCTGTAGGTTCTTCAGGACCCAAAGGAAGCCAGGGGGATCCAGGACGTCCAGGGGAACCTGGGCTTCCAGGTGCTCGGGGTTTGACAGGAAATCCTGGTGTTCAAGGTCCTGAAGGAAAACTTGGACCTTTGGGTGCGCCAGGGGAAGATGGCCGTCCAGGTCCTCCAGGCTCCATAGGAATCAGAGGGCAGCCCGGGAGCATGGGCCTTCCAGGCCCCAAAGGTAGCAGTGGTGACCCTGGGAAACCTGGAGAAGCAGGAAATGCTGGAGTTCCTGGGCAGAGGGGAGCTCCTGGAAAAGATGGTGAAGTTGGTCCTTCTGGTCCTGTGGGCCCGCCGGGTCTAGCTGGTGAAAGAGGAGAACAAGGACCTCCAGGCCCCACAGGTTTTCAGGGGCTTCCTGGTCCTCCAGGGCCTCCTGGAGAAGGTGGAAAACCAGGTGATCAAGGTGTTCCTGGAGATCCCGGAGCAGTTGGCCCGTTAGGACCTAGAGGAGAACGAGGAAATCCTGGGGAAAGAGGAGAACCTGGGATAACTGGACTCCCTGGTGAGAAGGGAATGGCTGGAGGACATGGTCCTGATGGCCCAAAAGGCAGTCCAGGTCCATCTGGGACCCCTGGAGATACAGGCCCACCAGGTCTTCAAGGTATGCCGGGAGAAAGAGGAATTGCAGGAACTCCTGGCCCCAAGGGTGACAGAGGTGGCATAGGAGAAAAAGGTGCTGAAGGCACAGCTGGAAATGATGGTGCAAGAGGTCTTCCAGGTCCTTTGGGCCCTCCAGGTCCGGCAGGTCCTACTGGAGAAAAGGGTGAACCTGGTCCTCGAGGTTTAGTTGGCCCTCCTGGCTCCCGGGGCAATCCTGGTTCTCGAGGTGAAAATGGGCCAACTGGAGCTGTTGGTTTTGCCGGACCCCAGGGTCCTGACGGACAGCCTGGAGTAAAAGGTGAACCTGGAGAGCCAGGACAGAAGGGAGATGCTGGTTCTCCTGGACCACAAGGTTTAGCAGGATCCCCTGGCCCTCATGGTCCTAATGGTGTTCCTGGACTAAAAGGTGGTCGAGGAACCCAAGGTCCGCCTGGTGCTACAGGATTTCCTGGTTCTGCGGGCAGAGTTGGACCTCCAGGCCCTGCTGGAGCTCCAGGACCTGCGGGACCCCTAGGGGAACCCGGGAAGGAGGGACCTCCAGGTCTTCGTGGGGACCCTGGCTCTCATGGGCGTGTGGGAGATCGAGGACCAGCTGGCCCCCCTGGTGGCCCAGGAGACAAAGGGGACCCAGGAGAAGATGGGCAACCTGGTCCAGATGGCCCCCCTGGTCCAGCTGGAACGACCGGGCAGAGAGGAATTGTTGGCATGCCTGGGCAACGTGGAGAGAGAGGCATGCCCGGCCTACCAGGCCCAGCGGGAACACCAGGAAAAGTAGGACCAACTGGTGCAACAGGAGATAAAGGTCCACCTGGACCTGTGGGGCCCCCAGGCTCCAATGGTCCTGTAGGGGAACCTGGACCAGAAGGTCCAGCTGGCAATGATGGTACCCCAGGACGGGATGGTGCTGTTGGAGAACGTGGTGATCGTGGAGACCCTGGGCCTGCAGGTCTGCCAGGCTCTCAGGGTGCCCCTGGAACTCCTGGCCCTGTGGGTGCTCCAGGAGATGCAGGACAAAGAGGAGATCCGGGTTCTCGGGGTCCTATAGGACCACCTGGTCGAGCTGGGAAACGTGGATTACCTGGACCCCAAGGACCTCGTGGTGACAAAGGTGATCATGGAGACCGAGGTGACAGAGGTCAGAAGGGCCACAGAGGCTTTACTGGTCTTCAGGGTCTTCCTGGCCCTCCTGGTCCAAATGGTGAACAAGGAAGTGCTGGAATCCCTGGACCATTTGGCCCAAGAGGTCCTCCAGGCCCAGTTGGTCCTTCAGGTAAAGAAGGAAACCCTGGGCCACTTGGGCCAATTGGACCTCCAGGTGTACGAGGCAGTGTAGGAGAAGCAGGACCTGAGGGCCCTCCTGGTGAGCCTGGCCCACCTGGCCCTCCGGGTCCCCCTGGCCACCTTACAGCTGCTCTTGGGGATATCATGGGGCACTATGATGAAAGCATGCCAGATCCACTTCCTGAGTTTACTGAAGATCAGGCGGCTCCTGATGACAAAAACAAAACGGACCCAGGGGTTCATGCTACCCTGAAGTCACTCAGTAGTCAGATTGAAACCATGCGCAGCCCCGATGGCTCGAAAAAGCACCCAGCCCGCACGTGTGATGACCTAAAGCTTTGCCATTCCGCAAAGCAGAGTGGTGAATACTGGATTGATCCTAACCAAGGATCTGTTGAAGATGCAATCAAAGTTTACTGCAACATGGAAACAGGAGAAACATGTATTTCAGCAAACCCATCCAGTGTACCACGTAAAACCTGGTGGGCCAGTAAATCTCCTGACAATAAACCTGTTTGGTATGGTCTTGATATGAACAGAGGGTCTCAGTTCGCTTATGGAGACCACCAATCACCTAATACAGCCATTACTCAGATGACTTTTTTGCGCCTTTTATCAAAAGAAGCCTCCCAGAACATCACTTACATCTGTAAAAACAGTGTAGGATACATGGACGATCAAGCTAAGAACCTCAAAAAAGCTGTGGTTCTCAAAGGGGCAAATGACTTAGATATCAAAGCAGAGGGAAATATTAGATTCCGGTATATCGTTCTTCAAGACACTTGCTCTAAGCGGAATGGAAATGTGGGCAAGACTGTCTTTGAATATAGAACACAGAATGTGGCACGCTTGCCCATCATAGATCTTGCTCCTGTGGATGTTGGCGGCACAGACCAGGAATTCGGCGTTGAAATTGGGCCAGTTTGTTTTGTGTAA

F TGTTCCTGGTCAACCTGGTGCTC

R ACCTTGCTGTCCTTGTAAACCCTG

199BP 55-60℃


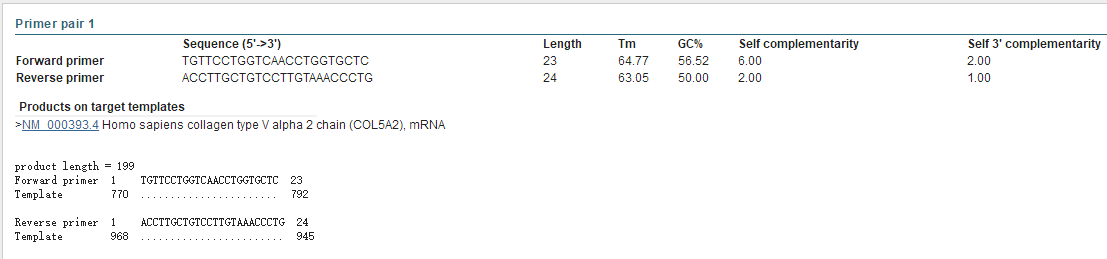


COL4A1

ATGGGGCCCCGGCTCAGCGTCTGGCTGCTGCTGCTGCCCGCCGCCCTTCTGCTCCACGAGGAGCACAGCCGGGCCGCTGCGAAGGGTGGCTGTGCTGGCTCTGGCTGTGGCAAATGTGACTGCCATGGAGTGAAGGGACAAAAGGGTGAAAGAGGCCTCCCGGGGTTACAAGGTGTCATTGGGTTTCCTGGAATGCAAGGACCTGAGGGGCCACAGGGACCACCAGGACAAAAGGGTGATACTGGAGAACCAGGACTACCTGGAACAAAAGGGACAAGAGGACCTCCGGGAGCATCTGGCTACCCTGGAAACCCAGGACTTCCCGGAATTCCTGGCCAAGACGGCCCGCCAGGCCCCCCAGGTATTCCAGGATGCAATGGCACAAAGGGGGAGAGAGGGCCGCTCGGGCCTCCTGGCTTGCCTGGTTTCGCTGGAAATCCCGGACCACCAGGCTTACCAGGGATGAAGGGTGATCCAGGTGAGATACTTGGCCATGTGCCCGGGATGCTGTTGAAAGGTGAAAGAGGATTTCCCGGAATCCCAGGGACTCCAGGCCCACCAGGACTGCCAGGGCTTCAAGGTCCTGTTGGGCCTCCAGGATTTACCGGACCACCAGGTCCCCCAGGCCCTCCCGGCCCTCCAGGTGAAAAGGGACAAATGGGCTTAAGTTTTCAAGGACCAAAAGGTGACAAGGGTGACCAAGGGGTCAGTGGGCCTCCAGGAGTACCAGGACAAGCTCAAGTTCAAGAAAAAGGAGACTTCGCCACCAAGGGAGAAAAGGGCCAAAAAGGTGAACCTGGATTTCAGGGGATGCCAGGGGTCGGAGAGAAAGGTGAACCCGGAAAACCAGGACCCAGAGGCAAACCCGGAAAAGATGGTGACAAAGGGGAAAAAGGGAGTCCCGGTTTTCCTGGTGAACCCGGGTACCCAGGACTCATAGGCCGCCAGGGCCCGCAGGGAGAAAAGGGTGAAGCAGGTCCTCCTGGCCCACCTGGAATTGTTATAGGCACAGGACCTTTGGGAGAAAAAGGAGAGAGGGGCTACCCTGGAACTCCGGGGCCAAGAGGAGAGCCAGGCCCAAAAGGTTTCCCAGGACTACCAGGCCAACCCGGACCTCCAGGCCTCCCTGTACCTGGGCAGGCTGGTGCCCCTGGCTTCCCTGGTGAAAGAGGAGAAAAAGGTGACCGAGGATTTCCTGGTACATCTCTGCCAGGACCAAGTGGAAGAGATGGGCTCCCGGGTCCTCCTGGTTCCCCTGGGCCCCCTGGGCAGCCTGGCTACACAAATGGAATTGTGGAATGTCAGCCCGGACCTCCAGGTGACCAGGGTCCTCCTGGAATTCCAGGGCAGCCAGGATTTATAGGCGAAATTGGAGAGAAAGGTCAAAAAGGAGAGAGTTGCCTCATCTGTGATATAGACGGATATCGGGGGCCTCCCGGGCCACAGGGACCCCCGGGAGAAATAGGTTTCCCAGGGCAGCCAGGGGCCAAGGGCGACAGAGGTTTGCCTGGCAGAGATGGTGTTGCAGGAGTGCCAGGCCCTCAAGGTACACCAGGGCTGATAGGCCAGCCAGGAGCCAAGGGGGAGCCTGGTGAGTTTTATTTCGACTTGCGGCTCAAAGGTGACAAAGGAGACCCAGGCTTTCCAGGACAGCCCGGCATGCCAGGGAGAGCGGGTTCTCCTGGAAGAGATGGCCATCCGGGTCTTCCTGGCCCCAAGGGCTCGCCGGGTTCTGTAGGATTGAAAGGAGAGCGTGGCCCCCCTGGAGGAGTTGGATTCCCAGGCAGTCGTGGTGACACCGGCCCCCCTGGGCCTCCAGGATATGGTCCTGCTGGTCCCATTGGTGACAAAGGACAAGCAGGCTTTCCTGGAGGCCCTGGATCCCCAGGCCTGCCAGGTCCAAAGGGTGAACCAGGAAAAATTGTTCCTTTACCAGGCCCCCCTGGAGCAGAAGGACTGCCGGGGTCCCCAGGCTTCCCAGGTCCCCAAGGAGACCGAGGCTTTCCCGGAACCCCAGGAAGGCCAGGCCTGCCAGGAGAGAAGGGCGCTGTGGGCCAGCCAGGCATTGGATTTCCAGGGCCCCCCGGCCCCAAAGGTGTTGACGGCTTACCTGGAGACATGGGGCCACCGGGGACTCCAGGTCGCCCGGGATTTAATGGCTTACCTGGGAACCCAGGTGTGCAGGGCCAGAAGGGAGAGCCTGGAGTTGGTCTACCGGGACTCAAAGGTTTGCCAGGTCTTCCCGGCATTCCTGGCACACCCGGGGAGAAGGGGAGCATTGGGGTACCAGGCGTTCCTGGAGAACATGGAGCGATCGGACCCCCTGGGCTTCAGGGGATCAGAGGTGAACCGGGACCTCCTGGATTGCCAGGCTCCGTGGGGTCTCCAGGAGTTCCAGGAATAGGCCCCCCTGGAGCTAGGGGTCCCCCTGGAGGACAGGGACCACCGGGGTTGTCAGGCCCTCCTGGAATAAAAGGAGAGAAGGGTTTCCCCGGATTCCCTGGACTGGACATGCCGGGCCCTAAAGGAGATAAAGGGGCTCAAGGACTCCCTGGCATAACGGGACAGTCGGGGCTCCCTGGCCTTCCTGGACAGCAGGGGGCTCCTGGGATTCCTGGGTTTCCAGGTTCCAAGGGAGAAATGGGCGTCATGGGGACCCCCGGGCAGCCGGGCTCACCAGGACCAGTGGGTGCTCCTGGATTACCGGGTGAAAAAGGGGACCATGGCTTTCCGGGCTCCTCAGGACCCAGGGGAGACCCTGGCTTGAAAGGTGATAAGGGGGATGTCGGTCTCCCTGGCAAGCCTGGCTCCATGGATAAGGTGGACATGGGCAGCATGAAGGGCCAGAAAGGAGACCAAGGAGAGAAAGGACAAATTGGACCAATTGGTGAGAAGGGATCCCGAGGAGACCCTGGGACCCCAGGAGTGCCTGGAAAGGACGGGCAGGCAGGACAGCCTGGGCAGCCAGGACCTAAAGGTGATCCAGGTATAAGTGGAACCCCAGGTGCTCCAGGACTTCCGGGACCAAAAGGATCTGTTGGTGGAATGGGCTTGCCAGGAACACCTGGAGAGAAAGGTGTGCCTGGCATCCCTGGCCCACAAGGTTCACCTGGCTTACCTGGAGACAAAGGTGCAAAAGGAGAGAAAGGGCAGGCAGGCCCACCTGGCATAGGCATCCCAGGGCTGCGAGGTGAAAAGGGAGATCAAGGGATAGCGGGTTTCCCAGGAAGCCCTGGAGAGAAGGGAGAAAAAGGAAGCATTGGGATCCCAGGAATGCCAGGGTCCCCAGGCCTTAAAGGGTCTCCCGGGAGTGTTGGCTATCCAGGAAGTCCTGGGCTACCTGGAGAAAAAGGTGACAAAGGCCTCCCAGGATTGGATGGCATCCCTGGTGTCAAAGGAGAAGCAGGTCTTCCTGGGACTCCTGGCCCCACAGGCCCAGCTGGCCAGAAAGGGGAGCCAGGCAGTGATGGAATCCCGGGGTCAGCAGGAGAGAAGGGTGAACCAGGTCTACCAGGAAGAGGATTCCCAGGGTTTCCAGGGGCCAAAGGAGACAAAGGTTCAAAGGGTGAGGTGGGTTTCCCAGGATTAGCCGGGAGCCCAGGAATTCCTGGATCCAAAGGAGAGCAAGGATTCATGGGTCCTCCGGGGCCCCAGGGACAGCCGGGGTTACCGGGATCCCCAGGCCATGCCACGGAGGGGCCCAAAGGAGACCGCGGACCTCAGGGCCAGCCTGGCCTGCCAGGACTTCCGGGACCCATGGGGCCTCCAGGGCTTCCTGGGATTGATGGAGTTAAAGGTGACAAAGGAAATCCAGGCTGGCCAGGAGCACCCGGTGTCCCAGGGCCCAAGGGAGACCCTGGATTCCAGGGCATGCCTGGTATTGGTGGCTCTCCAGGAATCACAGGCTCTAAGGGTGATATGGGGCCTCCAGGAGTTCCAGGATTTCAAGGTCCAAAAGGTCTTCCTGGCCTCCAGGGAATTAAAGGTGATCAAGGCGATCAAGGCGTCCCGGGAGCTAAAGGTCTCCCGGGTCCTCCTGGCCCCCCAGGTCCTTACGACATCATCAAAGGGGAGCCCGGGCTCCCTGGTCCTGAGGGCCCCCCAGGGCTGAAAGGGCTTCAGGGACTGCCAGGCCCGAAAGGCCAGCAAGGTGTTACAGGATTGGTGGGTATACCTGGACCTCCAGGTATTCCTGGGTTTGACGGTGCCCCTGGCCAGAAAGGAGAGATGGGACCTGCCGGGCCTACTGGTCCAAGAGGATTTCCAGGTCCACCAGGCCCCGATGGGTTGCCAGGATCCATGGGGCCCCCAGGCACCCCATCTGTTGATCACGGCTTCCTTGTGACCAGGCATAGTCAAACAATAGATGACCCACAGTGTCCTTCTGGGACCAAAATTCTTTACCACGGGTACTCTTTGCTCTACGTGCAAGGCAATGAACGGGCCCATGGCCAGGACTTGGGCACGGCCGGCAGCTGCCTGCGCAAGTTCAGCACAATGCCCTTCCTGTTCTGCAATATTAACAACGTGTGCAACTTTGCATCACGAAATGACTACTCGTACTGGCTGTCCACCCCTGAGCCCATGCCCATGTCAATGGCACCCATCACGGGGGAAAACATAAGACCATTTATTAGTAGGTGTGCTGTGTGTGAGGCGCCTGCCATGGTGATGGCCGTGCACAGCCAGACCATTCAGATCCCACCGTGCCCCAGCGGGTGGTCCTCGCTGTGGATCGGCTACTCTTTTGTGATGCACACCAGCGCTGGTGCAGAAGGCTCTGGCCAAGCCCTGGCGTCCCCCGGCTCCTGCCTGGAGGAGTTTAGAAGTGCGCCATTCATCGAGTGTCACGGCCGTGGGACCTGCAATTACTACGCAAACGCTTACAGCTTTTGGCTCGCCACCATAGAGAGGAGCGAGATGTTCAAGAAGCCTACGCCGTCCACCTTGAAGGCAGGGGAGCTGCGCACGCACGTCAGCCGCTGCCAAGTCTGTATGAGAAGAACATAA

F AGGATTTCCTGGTACATCTCTG

R GACATTCCACAATTCCATTTG

116bp 50-60℃


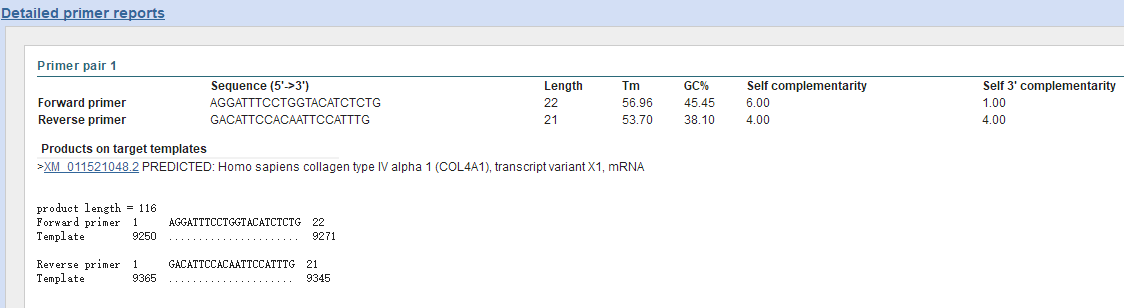


THBS2

ATGGTCTGGAGGCTGGTCCTGCTGGCTCTGTGGGTGTGGCCCAGCACGCAAGCTGGTCACCAGGACAAAGACACGACCTTCGACCTTTTCAGTATCAGCAACATCAACCGCAAGACCATTGGCGCCAAGCAGTTCCGCGGGCCCGACCCCGGCGTGCCGGCTTACCGCTTCGTGCGCTTTGACTACATCCCACCGGTGAACGCAGATGACCTCAGCAAGATCACCAAGATCATGCGGCAGAAGGAGGGCTTCTTCCTCACGGCCCAGCTCAAGCAGGACGGCAAGTCCAGGGGCACGCTGTTGGCTCTGGAGGGCCCCGGTCTCTCCCAGAGGCAGTTCGAGATCGTCTCCAACGGCCCCGCGGACACGCTGGATCTCACCTACTGGATTGACGGCACCCGGCATGTGGTCTCCCTGGAGGACGTCGGCCTGGCTGACTCGCAGTGGAAGAACGTCACCGTGCAGGTGGCTGGCGAGACCTACAGCTTGCACGTGGGCTGCGACCTCATAGACAGCTTCGCTCTGGACGAGCCCTTCTACGAGCACCTGCAGGCGGAAAAGAGCCGGATGTACGTGGCCAAAGGCTCTGCCAGAGAGAGTCACTTCAGGGGTTTGCTTCAGAACGTCCACCTAGTGTTTGAAAACTCTGTGGAAGATATTCTAAGCAAGAAGGGTTGCCAGCAAGGCCAGGGAGCTGAGATCAACGCCATCAGTGAGAACACAGAGACGCTGCGCCTGGGTCCGCATGTCACCACCGAGTACGTGGGCCCCAGCTCGGAGAGGAGGCCCGAGGTGTGCGAACGCTCGTGCGAGGAGCTGGGAAACATGGTCCAGGAGCTCTCGGGGCTCCACGTCCTCGTGAACCAGCTCAGCGAGAACCTCAAGAGAGTGTCGAATGATAACCAGTTTCTCTGGGAGCTCATTGGTGGCCCTCCTAAGACAAGGAACATGTCAGCTTGCTGGCAGGATGGCCGGTTCTTTGCGGAAAATGAAACGTGGGTGGTGGACAGCTGCACCACGTGTACCTGCAAGAAATTTAAAACCATTTGCCACCAAATCACCTGCCCGCCTGCAACCTGCGCCAGTCCATCCTTTGTGGAAGGCGAATGCTGCCCTTCCTGCCTCCACTCGGTGGACGGTGAGGAGGGCTGGTCTCCGTGGGCAGAGTGGACCCAGTGCTCCGTGACGTGTGGCTCTGGGACCCAGCAGAGAGGCCGGTCCTGTGACGTCACCAGCAACACCTGCTTGGGGCCCTCCATCCAGACACGGGCTTGCAGTCTGAGCAAGTGTGACACCCGCATCCGGCAGGACGGCGGCTGGAGCCACTGGTCACCTTGGTCTTCATGCTCTGTGACCTGTGGAGTTGGCAATATCACACGCATCCGTCTCTGCAACTCCCCAGTGCCCCAGATGGGGGGCAAGAATTGCAAAGGGAGTGGCCGGGAGACCAAAGCCTGCCAGGGCGCCCCATGCCCAATCGATGGCCGCTGGAGCCCCTGGTCCCCGTGGTCGGCCTGCACTGTCACCTGTGCCGGTGGGATCCGGGAGCGCACCCGGGTCTGCAACAGCCCTGAGCCTCAGTACGGAGGGAAGGCCTGCGTGGGGGATGTGCAGGAGCGTCAGATGTGCAACAAGAGGAGCTGCCCCGTGGATGGCTGTTTATCCAACCCCTGCTTCCCGGGAGCCCAGTGCAGCAGCTTCCCCGATGGGTCCTGGTCATGCGGCTCCTGCCCTGTGGGCTTCTTGGGCAATGGCACCCACTGTGAGGACCTGGACGAGTGTGCCCTGGTCCCCGACATCTGCTTCTCCACCAGCAAGGTGCCTCGCTGTGTCAACACTCAGCCTGGCTTCCACTGCCTGCCCTGCCCGCCCCGATACAGAGGGAACCAGCCCGTCGGGGTCGGCCTGGAAGCAGCCAAGACGGAAAAGCAAGTGTGTGAGCCCGAAAACCCATGCAAGGACAAGACACACAACTGCCACAAGCACGCGGAGTGCATCTACCTGGGCCACTTCAGCGACCCCATGTACAAGTGCGAGTGCCAGACAGGCTACGCGGGCGACGGGCTCATCTGCGGGGAGGACTCGGACCTGGACGGCTGGCCCAACCTCAATCTGGTCTGCGCCACCAACGCCACCTACCACTGCATCAAGGATAACTGCCCCCATCTGCCAAATTCTGGGCAGGAAGACTTTGACAAGGACGGGATTGGCGATGCCTGTGATGATGACGATGACAATGACGGTGTGACCGATGAGAAGGACAACTGCCAGCTCCTCTTCAATCCCCGCCAGGCTGACTATGACAAGGATGAGGTTGGGGACCGCTGTGACAACTGCCCTTACGTGCACAACCCTGCCCAGATCGACACAGACAACAATGGAGAGGGTGACGCCTGCTCCGTGGACATTGATGGGGACGATGTCTTCAATGAACGAGACAATTGTCCCTACGTCTACAACACTGACCAGAGGGACACGGATGGTGACGGTGTGGGGGATCACTGTGACAACTGCCCCCTGGTGCACAACCCTGACCAGACCGACGTGGACAATGACCTTGTTGGGGACCAGTGTGACAACAACGAGGACATAGATGACGACGGCCACCAGAACAACCAGGACAACTGCCCCTACATCTCCAACGCCAACCAGGCTGACCATGACAGAGACGGCCAGGGCGACGCCTGTGACCCTGATGATGACAACGATGGCGTCCCCGATGACAGGGACAACTGCCGGCTTGTGTTCAACCCAGACCAGGAGGACTTGGACGGTGATGGACGGGGTGATATTTGTAAAGATGATTTTGACAATGACAACATCCCAGATATTGATGATGTGTGTCCTGAAAACAATGCCATCAGTGAGACAGACTTCAGGAACTTCCAGATGGTCCCCTTGGATCCCAAAGGGACCACCCAAATTGATCCCAACTGGGTCATTCGCCATCAAGGCAAGGAGCTGGTTCAGACAGCCAACTCGGACCCCGGCATCGCTGTAGGTTTTGACGAGTTTGGGTCTGTGGACTTCAGTGGCACATTCTACGTAAACACTGACCGGGACGACGACTATGCCGGCTTCGTCTTTGGTTACCAGTCAAGCAGCCGCTTCTATGTGGTGATGTGGAAGCAGGTGACGCAGACCTACTGGGAGGACCAGCCCACGCGGGCCTATGGCTACTCCGGCGTGTCCCTCAAGGTGGTGAACTCCACCACGGGGACGGGCGAGCACCTGAGGAACGCGCTGTGGCACACGGGGAACACGCCGGGGCAGGTGCGAACCTTATGGCACGACCCCAGGAACATTGGCTGGAAGGACTACACGGCCTATAGGTGGCACCTGACTCACAGGCCCAAGACTGGCTACATCAGAGTCTTAGTGCATGAAGGAAAACAGGTCATGGCAGACTCAGGACCTATCTATGACCAAACCTACGCTGGCGGGCGGCTGGGTCTATTTGTCTTCTCTCAAGAAATGGTCTATTTCTCAGACCTCAAGTACGAATGCAGAGATATTTAA

F GCATCAAGGATAACTGCCCCCATCT

R TTCATTGAAGACATCGTCCCCATCA

290bp 55-65℃


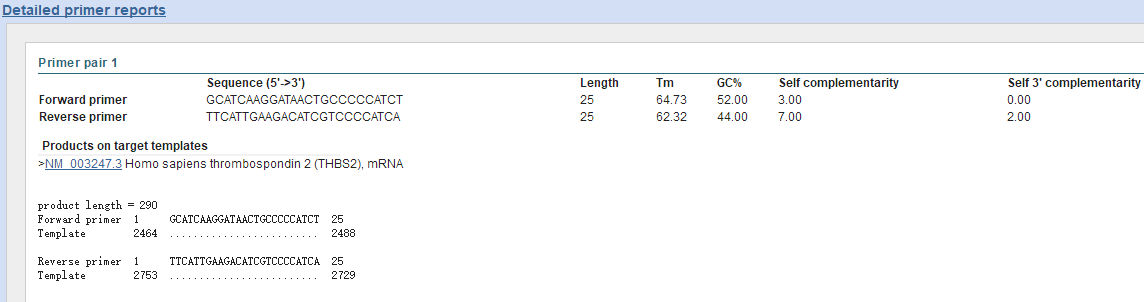


ITGA5

ATGGGGAGCCGGACGCCAGAGTCCCCTCTCCACGCCGTGCAGCTGCGCTGGGGCCCCCGGCGCCGACCCCCGCTGCTGCCGCTGCTGTTGCTGCTGCTGCCGCCGCCACCCAGGGTCGGGGGCTTCAACTTAGACGCGGAGGCCCCAGCAGTACTCTCGGGGCCCCCGGGCTCCTTCTTCGGATTCTCAGTGGAGTTTTACCGGCCGGGAACAGACGGGGTCAGTGTGCTGGTGGGAGCACCCAAGGCTAATACCAGCCAGCCAGGAGTGCTGCAGGGTGGTGCTGTCTACCTCTGTCCTTGGGGTGCCAGCCCCACACAGTGCACCCCCATTGAATTTGACAGCAAAGGCTCTCGGCTCCTGGAGTCCTCACTGTCCAGCTCAGAGGGAGAGGAGCCTGTGGAGTACAAGTCCTTGCAGTGGTTCGGGGCAACAGTTCGAGCCCATGGCTCCTCCATCTTGGCATGCGCTCCACTGTACAGCTGGCGCACAGAGAAGGAGCCACTGAGCGACCCCGTGGGCACCTGCTACCTCTCCACAGATAACTTCACCCGAATTCTGGAGTATGCACCCTGCCGCTCAGATTTCAGCTGGGCAGCAGGACAGGGTTACTGCCAAGGAGGCTTCAGTGCCGAGTTCACCAAGACTGGCCGTGTGGTTTTAGGTGGACCAGGAAGCTATTTCTGGCAAGGCCAGATCCTGTCTGCCACTCAGGAGCAGATTGCAGAATCTTATTACCCCGAGTACCTGATCAACCTGGTTCAGGGGCAGCTGCAGACTCGCCAGGCCAGTTCCATCTATGATGACAGCTACCTAGGATACTCTGTGGCTGTTGGTGAATTCAGTGGTGATGACACAGAAGACTTTGTTGCTGGTGTGCCCAAAGGGAACCTCACTTACGGCTATGTCACCATCCTTAATGGCTCAGACATTCGATCCCTCTACAACTTCTCAGGGGAACAGATGGCCTCCTACTTTGGCTATGCAGTGGCCGCCACAGACGTCAATGGGGACGGGCTGGATGACTTGCTGGTGGGGGCACCCCTGCTCATGGATCGGACCCCTGACGGGCGGCCTCAGGAGGTGGGCAGGGTCTACGTCTACCTGCAGCACCCAGCCGGCATAGAGCCCACGCCCACCCTTACCCTCACTGGCCATGATGAGTTTGGCCGATTTGGCAGCTCCTTGACCCCCCTGGGGGACCTGGACCAGGATGGCTACAATGATGTGGCCATCGGGGCTCCCTTTGGTGGGGAGACCCAGCAGGGAGTAGTGTTTGTATTTCCTGGGGGCCCAGGAGGGCTGGGCTCTAAGCCTTCCCAGGTTCTGCAGCCCCTGTGGGCAGCCAGCCACACCCCAGACTTCTTTGGCTCTGCCCTTCGAGGAGGCCGAGACCTGGATGGCAATGGATATCCTGATCTGATTGTGGGGTCCTTTGGTGTGGACAAGGCTGTGGTATACAGGGGCCGCCCCATCGTGTCCGCTAGTGCCTCCCTCACCATCTTCCCCGCCATGTTCAACCCAGAGGAGCGGAGCTGCAGCTTAGAGGGGAACCCTGTGGCCTGCATCAACCTTAGCTTCTGCCTCAATGCTTCTGGAAAACACGTTGCTGACTCCATTGGTTTCACAGTGGAACTTCAGCTGGACTGGCAGAAGCAGAAGGGAGGGGTACGGCGGGCACTGTTCCTGGCCTCCAGGCAGGCAACCCTGACCCAGACCCTGCTCATCCAGAATGGGGCTCGAGAGGATTGCAGAGAGATGAAGATCTACCTCAGGAACGAGTCAGAATTTCGAGACAAACTCTCGCCGATTCACATCGCTCTCAACTTCTCCTTGGACCCCCAAGCCCCAGTGGACAGCCACGGCCTCAGGCCAGCCCTACATTATCAGAGCAAGAGCCGGATAGAGGACAAGGCTCAGATCTTGCTGGACTGTGGAGAAGACAACATCTGTGTGCCTGACCTGCAGCTGGAAGTGTTTGGGGAGCAGAACCATGTGTACCTGGGTGACAAGAATGCCCTGAACCTCACTTTCCATGCCCAGAATGTGGGTGAGGGTGGCGCCTATGAGGCTGAGCTTCGGGTCACCGCCCCTCCAGAGGCTGAGTACTCAGGACTCGTCAGACACCCAGGGAACTTCTCCAGCCTGAGCTGTGACTACTTTGCCGTGAACCAGAGCCGCCTGCTGGTGTGTGACCTGGGCAACCCCATGAAGGCAGGAGCCAGTCTGTGGGGTGGCCTTCGGTTTACAGTCCCTCATCTCCGGGACACTAAGAAAACCATCCAGTTTGACTTCCAGATCCTCAGCAAGAATCTCAACAACTCGCAAAGCGACGTGGTTTCCTTTCGGCTCTCCGTGGAGGCTCAGGCCCAGGTCACCCTGAACGGTGTCTCCAAGCCTGAGGCAGTGCTATTCCCAGTAAGCGACTGGCATCCCCGAGACCAGCCTCAGAAGGAGGAGGACCTGGGACCTGCTGTCCACCATGTCTATGAGCTCATCAACCAAGGCCCCAGCTCCATTAGCCAGGGTGTGCTGGAACTCAGCTGTCCCCAGGCTCTGGAAGGTCAGCAGCTCCTATATGTGACCAGAGTTACGGGACTCAACTGCACCACCAATCACCCCATTAACCCAAAGGGCCTGGAGTTGGATCCCGAGGGTTCCCTGCACCACCAGCAAAAACGGGAAGCTCCAAGCCGCAGCTCTGCTTCCTCGGGACCTCAGATCCTGAAATGCCCGGAGGCTGAGTGTTTCAGGCTGCGCTGTGAGCTCGGGCCCCTGCACCAACAAGAGAGCCAAAGTCTGCAGTTGCATTTCCGAGTCTGGGCCAAGACTTTCTTGCAGCGGGAGCACCAGCCATTTAGCCTGCAGTGTGAGGCTGTGTACAAAGCCCTGAAGATGCCCTACCGAATCCTGCCTCGGCAGCTGCCCCAAAAAGAGCGTCAGGTGGCCACAGCTGTGCAATGGACCAAGGCAGAAGGCAGCTATGGCGTCCCACTGTGGATCATCATCCTAGCCATCCTGTTTGGCCTCCTGCTCCTAGGTCTACTCATCTACATCCTCTACAAGCTTGGATTCTTCAAACGCTCCCTCCCATATGGCACCGCCATGGAAAAAGCTCAGCTCAAGCCTCCAGCCACCTCTGATGCCTGA

F TAATACCAGCCAGCCAGGAGTG

R TGTCAAATTCAATGGGGGTGC

95bp 50-60℃


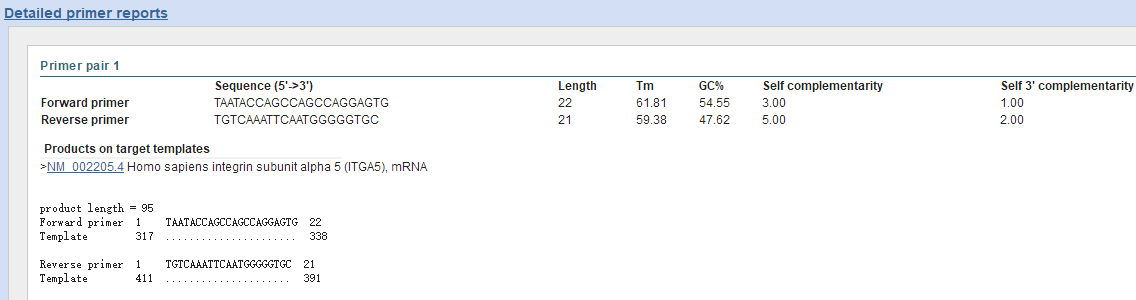


注：

1. 引物方向均为5’-3’。
2. 引物退火温度请在推荐范围内优化。
3. 截图为primer-blast结果。
